# DeepPathway: predicting pathway expression from histopathology images

**DOI:** 10.1093/bioinformatics/btag643

**Published:** 2026-08-26

**Authors:** Muhammad Ahtazaz Ahsan, Karen Piper Hanley, Martin Fergie, Claire O’leary, Gerben Borst, Federico Roncaroli, Fayyaz Minhas, Magnus Rattray, Mudassar Iqbal, Syed Murtuza Baker

**Affiliations:** Faculty of Biology, Medicine and Health, The University of Manchester, Manchester, M13 9PT, United Kingdom; Faculty of Biology, Medicine and Health, The University of Manchester, Manchester, M13 9PT, United Kingdom; Faculty of Biology, Medicine and Health, The University of Manchester, Manchester, M13 9PT, United Kingdom; Faculty of Biology, Medicine and Health, The University of Manchester, Manchester, M13 9PT, United Kingdom; Faculty of Biology, Medicine and Health, The University of Manchester, Manchester, M13 9PT, United Kingdom; Faculty of Biology, Medicine and Health, The University of Manchester, Manchester, M13 9PT, United Kingdom; Department of Computer Science, University of Warwick, Coventry, CV4 7AL, United Kingdom; Faculty of Biology, Medicine and Health, The University of Manchester, Manchester, M13 9PT, United Kingdom; Faculty of Biology, Medicine and Health, The University of Manchester, Manchester, M13 9PT, United Kingdom; Faculty of Biology, Medicine and Health, The University of Manchester, Manchester, M13 9PT, United Kingdom; Advanced Bioinformatics and Computational, University of Manchester, Manchester, M13 9PT, United Kingdom

## Abstract

**Motivation:**

Spatial transcriptomics (ST) technologies provide spatially resolved gene expression along with image data, allowing the integrative analysis of complex tissue microenvironment. Despite their potential, the widespread adoption of ST remains limited due to high costs, and methodological challenges in data acquisition. Thus, there have been recent efforts to develop deep learning methods for inferring spatial gene expression from much cheaper and easily available haematoxylin and eosin (H&E) images. These methods demonstrate promising results in reconstructing transcriptomic landscapes within tissue sections. While existing approaches focus on gene-level predictions, biological processes are often regulated at the pathway level through coordinated activity among functionally related genes.

**Results:**

We present DeepPathway, a contrastive learning-based approach trained on ST data to predict pathway expression from H&Es. We compute input pathway expression by summarizing the expression of constituent genes using established pathway definitions. We evaluate the performance of DeepPathway on multiple cancer datasets and validate it on the H&E images from The Cancer Genome Atlas (TCGA) clearly differentiating certain pathway activities in normal and tumour tissue regions. Finally, we apply our method to predict hypoxia signatures using H&Es of brain tumour samples where hypoxia staining with pimonidazole was available as ground truth.

**Code availability:**

Implementation code for DeepPathway is available at https://doi.org/10.5281/zenodo.21100191 and at GitHub repository: https://github.com/aahsan045/DeepPathway.

## 1 Introduction

Tissues in multi-cellular organisms consist of diverse cell populations that coordinate their functions by spatially interacting with each other (Zhuang 2021). Single-cell RNA sequencing (scRNAseq) technologies allow high-throughput gene expression profiling to study individual cellular activity but lack the spatial context. To address this limitation, spatial transcriptomics (ST) (Marx 2021) technologies, such as the Visium platform by 10x Genomics and Slide-seq (Rodriques *et al.* 2019) have been developed which retain the spatial context of cells while capturing gene expression data. ST platforms often include co-registered Haematoxylin and Eosin (H&E)-stained images providing a view of tissue architecture. The integration of pathological, ST and molecular data enables researchers to accurately analyse tissue regions that can be relevant to diagnosis, disease progression or are driven by molecular pathways that can be targeted with effective treatment modalities (Zheng and Fang 2022).

Although ST provides detailed molecular insights, it is an expensive and laborious technique that requires unique expertise. In contrast, tissue scanned H&E-stained images are cheap and readily available, from routine diagnostic provision. Recent advances in deep learning (DL)-based methods have enabled the prediction of spatial gene expression directly from histology images (He *et al.* 2020, Bae *et al.* 2021, Monjo *et al.* 2022, Jia *et al.* 2023, Xie *et al.* 2023, Li *et al.* 2024, Min *et al.* 2024, Pizurica *et al.* 2024, Xiao *et al.* 2024, Shi *et al.* 2025, Wang *et al.* 2025b). These models are trained on ST datasets and then used to infer gene expression patterns from H&E images alone. For example, ST-Net (He *et al.* 2020) utilises DenseNet-121 (pretrained on ImageNet) for extracting features from H&E images and predicts expression levels for 250 genes. Similarly, BLEEP (Bimodal Embedding for Expression Prediction), proposed by Xie *et al.* (2023), employs a bimodal contrastive learning framework to learn a shared embedding space between H&E patches and corresponding gene expression profiles. Despite being trained on multiple genes, these methods tend to predict only a limited subset of disease-relevant genes with reasonable accuracy.

Normal cellular functions result from the coordination of complex, hierarchical gene networks (https://www.genome.gov/about-genomics/fact-sheets/Biological-Pathways-Fact-Sheet). The analysis of such networks and pathways rather than individual genes reflects more closely the tissue homeostasis in normal and pathological conditions. To translate a set of individual gene expression profiles into a pathway expression profile at cellular or spot level resolution, methods such as AUCell (Aibar *et al.* 2017) or Ucell (Andreatta and Carmona 2021) can be utilised. This provides a way to transform thousands of genes into a handful of relevant pathways which could be more accurately and efficiently learned and could have more translational impact in identifying biomarkers for diagnosing certain disease phenotypes.

With the aim to predict the pathway expression from H&E images, we develop a DL-based approach called DeepPathway, adapted from BLEEP (Xie *et al.* 2023) which is a contrastive learning framework for learning a joint embedding space of expression and image modality. However, BLEEP has not been developed for pathway expression prediction problem. Also, it requires access to ST training data (or its embeddings) to infer the expression of test histology image, somewhat limiting its practical deployment. Hence, we introduce a novel two-stage model for pathway expression prediction replacing BLEEP’s inference mechanism with a deep neural network (DNN) that directly predicts pathway expression from H&E features. The key contributions of this work are given below.

1. We demonstrate the effectiveness of pathway expression prediction over gene expression prediction using prostate cancer data. For this we introduce a new approach adapted from an existing bimodal contrastive learning framework to predict the pathway expression from H&E images.
2. We propose a two-stage learning framework, DeepPathway, to predict pathway expression directly from H&E image.
3. We apply and evaluate our approach on a larger prostate carcinoma ST dataset. Additionally, we show the application of the proposed method on the H&Es of the prostate tumour samples from The Cancer Genome Atlas (TCGA).
4. Finally, we use DeepPathway to identify hypoxic regions in brain tumour patient samples using only H&E images with hypoxia specific staining with pimonidazole.

## 2 Materials and methods

### 2.1 Approach overview

**Figure 1:**
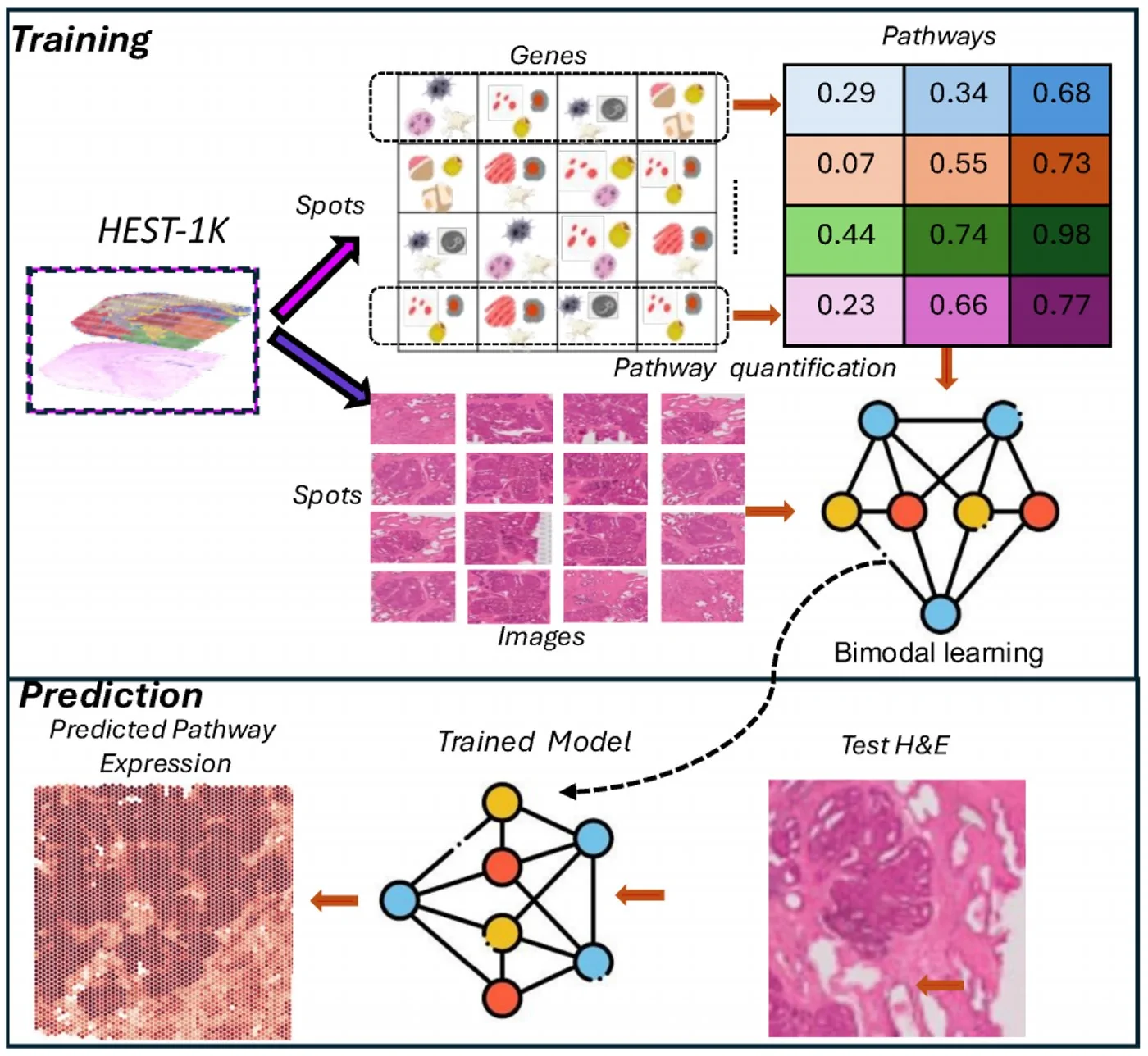
Overview of the proposed method (DeepPathway); Spot-level gene expression and H&E image patches are acquired from HEST-1k database. Gene expression data is summarized into spots × pathway expression matrix using pathway quantification with UCell (Andreatta and Carmona 2021). In the training part, a deep learning model is trained using bimodal contrastive learning on spot-level image patches and pathway expression data. At the inference time, only H&E image is passed to the trained model for predicting pathway expression.

A brief overview of DeepPathway is presented in Fig. 1 which shows as input paired H&E images and gene expression data coming from ST experiments. After initial gene expression and H&E image processing, using literature-based pathway definitions (e.g. MSigDB hallmark), we transformed the original spot-by-gene matrix into a spot-by-pathway matrix using UCell (Andreatta and Carmona 2021). We train DeepPathway on paired image patch and pathway expression data to learn a common embedding representation via contrastive learning. Finally, we use trained DeepPathway to predict the pathway expression of test H&E images.

We organised the work reported here into three interconnected approaches, which are briefly described as follows. (i) At first, we used BLEEP as the baseline method to perform and compare gene expression prediction followed by conversion into pathway expression versus direct pathway expression prediction highlighting the utility of our approach. (ii) In the second approach, we improved image feature representations by integrating outputs from a trainable BLEEP image encoder with features from the non-trainable H&E image foundation model, *H-optimus-0* (Saillard *et al.* 2024) (used as a prior for improved learning). (iii) Finally, we replace the inference part of BLEEP with training a deep neural network (DNN) model for improved deployability. This results in a proposed two-stage learning framework in which, first a model having an image encoder with optimus-and expression encoder is trained using contrastive learning (stage-1). In stage-2, we train a DNN model on image features extracted from the image models used in the stage-1 to predict the pathway expression directly (see Fig. 3A).

Throughout all stages, we leveraged the bimodal contrastive learning framework introduced in BLEEP (Xie *et al.* 2023) that was originally developed for predicting spatial gene expression from H&E images. The model takes randomly shuffled batch of samples for training. The main objective is to learn the same-sized embedding space between matching image-expression pairs. As model training continues, the model improves its performance by capturing meaningful cross-modal similarities while pushing apart dissimilar pairs, thereby enhancing the modality alignment. In the original BLEEP inference setup, test image embeddings are compared to those from the training set, and the pathway expression is estimated by averaging the outputs from the top k closest matches. In contrast, our two-stage framework eliminated the need for training data (or embeddings) at inference time and pathway expressions are predicted directly from the image features using the trained DNN.

### 2.2 Bimodal contrastive learning for pathway expression prediction

Contrastive learning (CL) (Chen *et al.* 2020, Khosla *et al.* 2020) is a computational method applied to learn meaningful semantic representation by pulling similar samples closer to each other while pushing dissimilar samples apart. This idea has been extended to bimodal data such as image-text, to learn a joint feature representation across modalities. Additionally, it is widely used for developing DL-based applications such as image-caption generation (Dai and Lin 2017) or text-to-image generation (Zhang *et al.* 2021). Adapting the idea of CL from BLEEP, given a spot (a spatially barcoded area where gene expression is profiled) $S=\{X_{\mathrm{img}},\;X_{\mathrm{expr}}\}$, that consisted of paired image-expression profile, a learnable image encoder ($f_{\theta }(X_{\mathrm{img}})$) and an expression encoder ($f_{\phi }(X_{\mathrm{expr}})$) are used to produce embeddings $Z_{\mathrm{img}}$ and $Z_{\mathrm{expr}}$, respectively. We then add the positional encoding of the corresponding spot location coordinates (x,y) into both embeddings by adapting idea of sinusoidal positional encoding ($f_{PE}$ (x, y)) as proposed for transformers (Vaswani *et al.* 2017). Finally, we calculate the contrastive loss using Equation 1.


(1) $$\begin{array}{l} Z_{\mathrm{img}}=f_{\theta }(X_{\mathrm{img}})+f_{PE}(x,y),\\ Z_{\mathrm{expr}}=f_{\phi }(X_{\mathrm{expr}})+f_{PE}(x,y),\\ \mathrm{logits}=Z_{\mathrm{img}}\cdot Z_{\mathrm{expr}}^{T},\\ \mathrm{targets}=\mathrm{Softmax}(\frac{Z_{\mathrm{img}}\cdot Z_{\mathrm{img}}^{T}+Z_{\mathrm{expr}}\cdot Z_{\mathrm{expr}}^{T}}{2}),\\ \mathrm{los}s_{CL}=\frac{CE(\mathrm{logits},\mathrm{targets})+CE(\mathrm{logit}s^{T},\mathrm{target}s^{T})}{2} \end{array}$$

where *logits* is the paired similarity vector between both modalities treated as a ground truth and *targets* is the averaged internal similarity between both modalities followed by *softmax*. Then cross-entropy (CE) is applied between them to calculate the image and expression loss followed by taking their mean for producing contrastive loss. The final loss (\L) is calculated by adding λ scaled $L_{1}$ regularization to both image and expression embeddings,


(2) $$L=\mathrm{los}s_{CL}+\lambda ({\left\|Z_{\mathrm{img}}\right\|}_{1}+{\left\|Z_{\mathrm{expr}}\right\|}_{1})\;.$$

### 2.3 Dataset

We obtained publicly available 10X Visium datasets from the HEST-1k database (Jaume *et al.* 2025), which provides uniformly processed high quality images, gene expression profile, metadata for each ST sample, covering multiple organs and diseases. Specifically in HEST-1k, all whole-slide images (WSIs) are normalized and transformed to generic Pyramidal-based TIFF image objects that can be integrated with computational tools such as OpenSlide (Goode *et al.* 2013) or image viewers like QUPath (Humphries *et al.* 2021). In this work, we used two different human prostate cancer datasets from HEST-1k, one we refer to as small prostate cancer dataset (Jaume *et al.* 2025) and other as large prostate cancer dataset (Erickson *et al.* 2022) hereafter. The large data from prostate adenocarcinoma consisted of two patients, patient 1 has 8 samples while patient 2 has 15 specimens, all diagnosed as Gleason 4 + 3 score group 3. Additionally, we used skin cancer dataset (Ji *et al.* 2020), consisted of 4 ST samples. For prediction of hypoxia pathway, we used 5 glioblastoma multiforme (GBM) cancer ST samples from Ravi *et al.* (2022). Details about total number of samples used for each dataset, total number of spots left after preprocessing, and image resolution of whole slide images (WSIs) for each dataset can be seen in Table S1.

### 2.4 Data processing

For each dataset, we performed gene and spot filtering to remove genes (expressed in ≤ 10 spots) and spots (having ≤ 500 genes expressed), respectively. Then we selected the top 10,000 highly variable genes (HVG) from each sample. The reason for selecting a large number of HVGs is to keep the maximum overlap between each sample’s genes with the pathway-specific genes. For pathways definitions, we used the Molecular Signature Database (MSigDB) hallmark collection (Liberzon *et al.* 2015), which is commonly used for cancer studies. It consists of 50 pathway definitions with the unique number of genes equal to 4383. After acquiring the spot-level gene count data, we performed the total normalization (to deal with different sequencing depths) followed by the *log1p* transformation (to reduce data skewness) using *scanpy* (Wolf *et al.* 2018).

We performed the H&E image processing on the spots left after gene expression processing. For each dataset, we cropped the image patch of 224 × 224 pixels centered at each spot’s location using the given resolution of microns per pixel through the TIA toolbox (Pocock *et al.* 2022). We removed spots where more than 75% of the image represented the background, i.e. having pixel value in all three color channels more than 200. For TCGA and GBM data, we cropped patches as follows. Starting from the top-left corner, i.e. at (0,0) location of the image, non-overlapping patches of 224×224 were taken and passed to the image processing pipeline to filter out non-informative patches (as mentioned above). For GBM samples (Ravi *et al.* 2022), we applied Reinhard stain normalization to obtain the same histology colors distribution for smooth model training. During inference, same image processing is applied to predict pathway expression while their locations were preserved for visualization. Additionally, we have integrated the SpatialData (Marconato *et al.* 2025) in DeepPathway’s code, which provides a Python-based implementation of handling spatial omics data data, for unified data storage and visualization.

### 2.5 Pathway expression quantification

We used UCell (Andreatta and Carmona 2021) to quantify the pathway expression given the gene expression measurements and pathway definitions from MSigDB hallmark. UCell is a recent pathway quantification method which is time and memory efficient compared to AUCell. It uses Mann-Whitney statistics to compare the rank distribution of genes in a pathway versus all other genes. It is robust to differences in dataset compositions as it only considers relative gene expression in a given spot/cell and avoids an arbitrary threshold which is the case in AUCell. In this method, genes in each spot are ranked (from high to low) based on their expression values. Then, for a $j^{th}$ spot, the UCell score ($U_{j}$) for a pathway composed of *n* genes is calculated using Equation 3.


(3) $$U_{j}=1-\frac{\left(\sum_{i=0}^{n}r_{ij}-\frac{n(n+1)}{2}\right)}{n.R_{\max}-\frac{n(n+1)}{2}}$$

where $r_{ij}$ is the rank value of $i^{th}$ the gene in the $j^{th}$ spot. The $R_{\max}$ is the maximum rank value used to drop the uninformative tail of genes with low expression. We selected the $R_{\max}$ value for each dataset independently using the following method. For a given spot within a sample, we sorted the gene expression from highest to lowest values and obtained the rank index of the first zero value. We repeated this for all spots across all samples and took the median to obtain the final $R_{\max}$ threshold. The reason for this approach to select $R_{\max}$ threshold is to give constant rank value for all non-expressed genes to mitigate the long tail of bottom ranking genes. By following this procedure, we found the $R_{\max}$ value of 4276, 1926, 1954, 1265, and 2106 for small prostate, large prostate cancer patient 1, large prostate cancer patient 2, skin cancer, and GBM cancer data, respectively. Also, we selected only those pathways that contained at least 70% of the genes overlapping with the ST expression dataset genes (that are union of all samples’ genes in the dataset) and contain minimum 20 genes. By using this threshold, we found the number of pathways for small, large prostate cancer, and skin cancer datasets to be 50, 47, and 44, respectively. For GBM data, we used 7 brain tumor specific pathway definitions. In this study, we used UCell-derived pathway expression values as ground truth, however, it should not be interpreted as absolute experimental ground truth, but rather as rank-based pathway activity estimates derived from the ST measurements.

### 2.6 Model training and validation

We assessed the performance of each model using leave-one-out cross validation, where one sample from the data is held out for testing while remaining tissue samples are divided into spot-level training and validation data. We used ResNet18 for pathway prediction task in DeepPathway while we used ResNet50 for both gene and pathway expression prediction for the sake of fair comparison with BLEEP model. Hyperparameters with their values used in this study are shown in Table S2.

## 3 Results

### 3.1 Bimodal contrastive learning for gene versus pathway expression prediction on prostate cancer data

**Figure 2:**
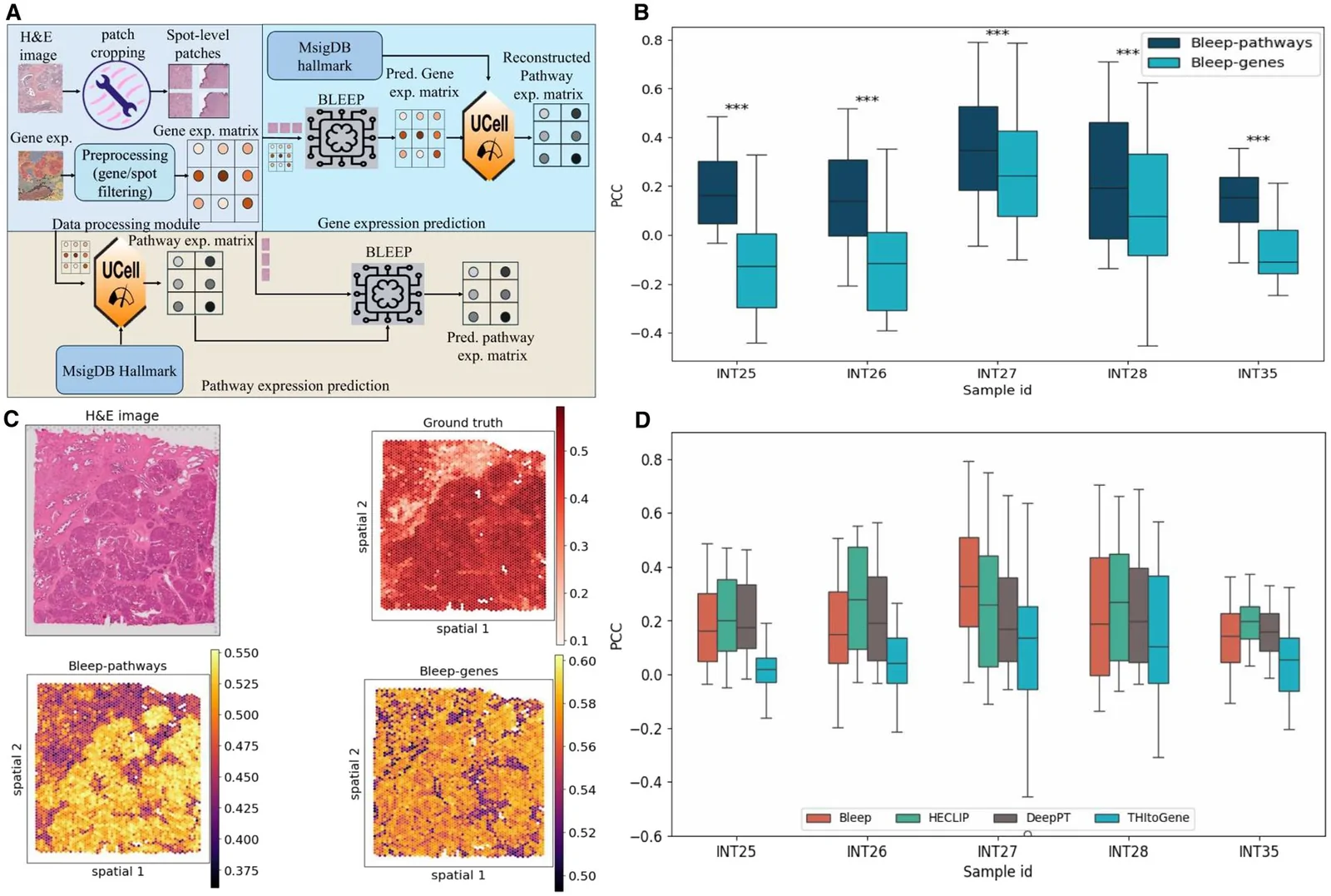
(A) Overview of the baseline method where spot-level data is initially processed (data processing module). Then, BLEEP is first trained on paired H&E images and gene expression data to predict the gene expression. Then predicted gene expression is passed to the UCell for obtaining the pathway expression. Whereas in pathway expression prediction (bottom half), gene expression matrix is first converted into pathway expression matrix followed by training BLEEP to predict the pathway expression. Finally, the pathway expression matrix from gene expression prediction (BLEEP-genes) is compared with the predicted pathway expression (BLEEP-pathways) obtained from pathway expression prediction. (B) PCC distribution plot of BLEEP-pathways versus BLEEP-genes for all samples where “***” symbol shows the magnitude of *P*-value (<0.001) calculated from paired t-test of two PCC distributions. (C) Spatial visualization of ground truth pathway expression from UCell, predicted *Androgen response* pathway expression from BLEEP-pathways and BLEEP-genes having PCC value of 0.63 and 0.13, respectively, with H&E image (INT28). (D) PCC distribution plot showing comparison of BLEEP with other existing methods in predicting pathway expression. All experiments were conducted on small prostate cancer dataset.

To being with, we applied BLEEP to perform gene expression prediction followed by conversion to pathway expression and direct pathway expression prediction on the small prostate carcinoma dataset to establish the utility of pathway-level prediction while using the same underlying contrastive learning approach. At first, spot-level paired gene and H&E image data is processed and BLEEP having ResNet50 as an image encoder is trained on paired data to predict gene expression. This trained model is then used to predict gene expression for test sample, which is then converted into pathway expression by Ucell, utilising MSigDB hallmark pathway definitions (we call this approach “BLEEP-genes”). In parallel, we passed the processed gene expression data along with the MSigDB pathway definitions to UCell to obtain pathway expression data. Then, we trained BLEEP using same ResNet50 model (image encoder) with paired pathway expression data to predict the pathway expression (we call this approach “BLEEP-pathways”). We used ResNet50 only here in BLEEP-pathways to make a fair comparison with BLEEP-genes. Finally, reconstructed pathway expression obtained from BLEEP-genes and predicted pathway expression from BLEEP-pathways are compared (Fig. 2A) by computing the PCC between the ground truth pathway expression of the test sample—and both the reconstructed and predicted pathway expressions. Overall, BLEEP-pathways clearly outperformed BLEEP-genes (Fig. 2B). For example, in the case of Androgen Response (Fig. 2C), BLEEP-pathways has PCC of 0.63 while BLEEP-genes with 0.13. On the other hand for some pathways, BLEEP-pathways and BLEEP-genes performed similarly, for example Epithelial Mesenchymal Transition (EMT) pathway, which plays a crucial role in tumour metastasis (Mittal 2018), in samples “INT27” and “INT28,” have achieved PCC values of around 0.79 and 0.55, for both the approaches, respectively. This potentially depicts that genes in the EMT pathway are well correlated with the histological microscopic features, helping predicting the genes and its constituent pathway very well in both scenarios. Additionally, we computed the PCC between top 5 highly expressed and highly variable pathways from BLEEP-genes and BLEEP-pathways (Fig. S1). It can be seen that BLEEP-pathways is consistent with higher PCC values across all samples, which depict that it is more meaningful to perform direct pathway expression prediction as it leverages the advantage of focusing on aggregate biological signal rather than individual gene fluctuations. Furthermore, for the sake of a fair and thorough comparison, we evaluated BLEEP-genes and BLEEP-pathways on ResNet18 as an image encoder (Fig. S2), showing similar results of BLEEP-pathways performing overall better in most of the samples compared to BLEEP-genes.

In order to select the most suitable gene expression prediction model for adaptation to pathway expression prediction as well as using it as baseline for further improvements, we performed comparison of BLEEP with existing methods, i.e. HECLIP (Wang *et al.* 2025b), DeepPT (Hoang *et al.* 2023), and THItoGene (Jia *et al.* 2023). DeepPT and THItoGene are selected from top performing gene expression prediction methods, as reported in the benchmarking study (Wang *et al.* 2025a). These were adaptable to pathway expression prediction task given their underlying model architectures while others like Hist2ST (Zeng *et al.* 2022) and HGGEP (Li *et al.* 2024) are not suitable for pathway expression prediction because they are trained using count-based objectives with zero-inflated negative binomial loss. Additionally, HGGEP uses a graph neural network to model all spatial locations from a slide within each training instance, limiting its scalability for large 10x Visium datasets. We also included recently published method HECLIP in the comparison as its architecture is very similar to BLEEP model. We evaluated each model on small prostate carcinoma data and the PCC distribution of each test sample is shown in Fig. 2D. Our results show that BLEEP outperformed other methods while achieved similar performance as HECLIP. Thus, we preferred BLEEP over other methods and built our proposed model (explained in next section) using BLEEP as a baseline model.

### 3.2 Improved pathway expression prediction model deployability via two-stage learning framework

**Figure 3:**
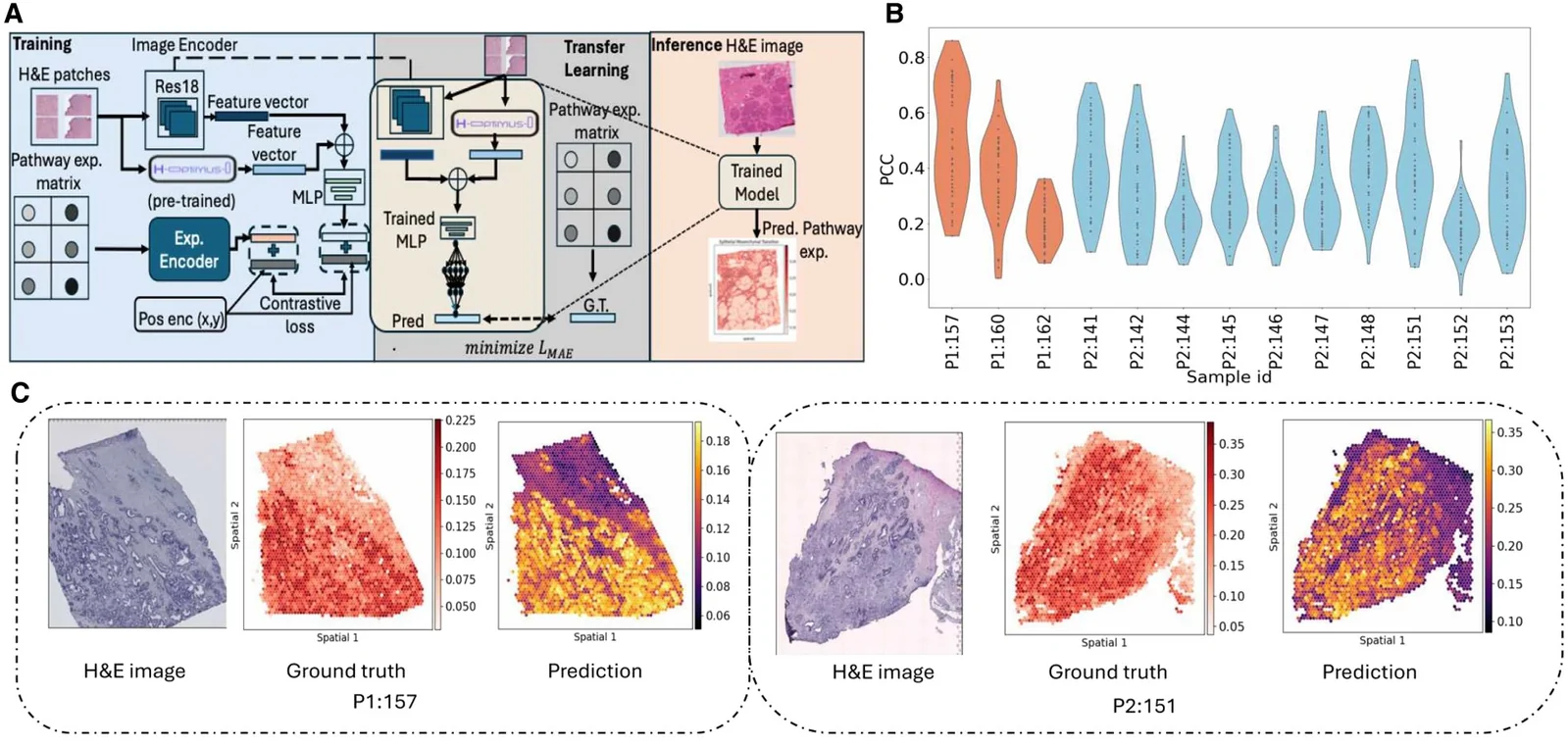
(A) Overview of DeepPathway: the proposed two-stage learning framework. At first, ResNet18 is trained on combined image features that are obtained from ResNet18 and H-optimus-0 foundation model using bimodal contrastive learning. In the second stage, a DNN is trained on image features extracted from trained stage-1 model to predict pathway expression by minimizing MAE. During inference time, only H&E image is used to predict pathway expression. (B) PCC distribution plot of test samples from patient 1 (P1) and patient 2 (P2) when the model is trained using the training samples from both patients. (C) Test H&E image and spatial visualization of Androgen Response pathway from P2 (best predicted) and mTORC1 Signaling pathway from P1 (second best predicted, just to show different pathway) shows the ground truth pathway expression, obtained from UCell, alongside the predicted pathway expression from the trained model (PCC = 0.79 for both).

One of the limitations of the BLEEP model is that it requires the training data (latent projections) to infer the pathway expression for the test data. Considering future deployability of these models, we propose a two-stage learning framework (Fig. 3A); we trained ResNet18 as an image encoder with *H-optimus-0* (non-trainable) using contrastive learning (stage-1) followed by training a DNN model on features obtained from stage-1 as an input to predict pathway expression. This approach minimizes the mean absolute error (MAE) between the ground truth and prediction, which is calculated as $\mathrm{MAE}(y,y^{\prime })=\frac{\sum_{i=1}^{N}{\left\|y-y^{\prime }\right\|}_{1}}{N}$, where *y* is ground truth pathway expression obtained from UCell and $y^{\prime }$ is the predicted pathway expression for a batch of *N* samples. Additionally, here we did not add the positional encodings (PE), as we are obtaining the image features from the trained model which has already learned the spatial similarities between those modalities. During inference, we used the trained ResNet18, *H-optimus-0* (stage 1), and the trained DNN model (stage 2) to predict the patch-level pathway expression. Beside improved deployability, adding a DNN in the two-stage architecture makes the model lightweight for prediction and open ups the way of developing transfer learning based applications in future. We did an ablation study evaluating different combinations of the proposed two-stage model, i.e. BLEEP with Optimus, BLEEP with DNN and ResNet18 with Optimus and DNN (which is our final model and we call it as DeepPathway) on small prostate cancer data where DeepPthway was the best performing among all (Fig. S3). Subsequently, we applied DeepPathway on large human prostate carcinoma data, classified as Gleason 4 + 3, grade group 3 (Erickson *et al.* 2022) using two approaches, which are described as follows. Within the large human prostate data, we picked two patients with largest number of samples and used 5 random samples from each (a total of 10 samples) as training data for DeepPathway, and then performed testing on remaining 13 samples of both patients (mixed patients’ samples). Each patient was processed independently using their own ST data for fair comparison. In second approach, we used patient specific data using leave-one-patient-out cross validation, i.e. train on P1, test on P2, and vice versa. We evaluated model’s performance stage-wise, i.e. stage-1 followed by stage-2 using both approaches separately to show the model’s robustness and generalisation. We have also performed an ablation study of DeepPathway with and without PE (see Fig. S4, for different experimental settings) and retain adding PE in stage-1 only in our final model. Results for both approaches are described below.

#### 3.2.1 Evaluation on mixed patient’s samples

The results for stage-1 model using mixed patient samples are shown in Fig. S5 where variation in PCC distributions depicts differences in model performance across different patient samples, potentially pointing to intrinsic heterogeneity within the tissue which could influence model’s performance. Additionally, we evaluated stage-1 approach on small prostate cancer data (Fig. S6). These results show that our proposed approach achieved improved results in terms of pathway expression prediction with the inclusion of the *H-optimus-0* foundation model.

For stage-2, i.e. training a DNN for direct prediction with mixed patient data, PCC distribution between ground truth and predicted pathway expression is shown in Fig. 3B. A similar trend of PCC distribution is observed when compared to Fig. S3B, as the bimodal contrastive loss model remained same in both approaches. We selected two different pathways (selected from the top predicted), i.e. mTORC1 Signaling pathway from P1 (ID = 157) and Androgen Response pathway from P2 (ID = 151) with the same PCC value of 0.79, are visualised in Fig. 3C. Although, Androgen Response was top predicted in P1:157 with PCC of 0.86. This shows that our proposed approach not only aligns the image-expression modality using contrastive learning but also learned a meaningful association of image features with pathway expression through a simple DNN model.

#### 3.2.2 Evaluation using leave-one-patient-out cross validation

**Figure 4:**
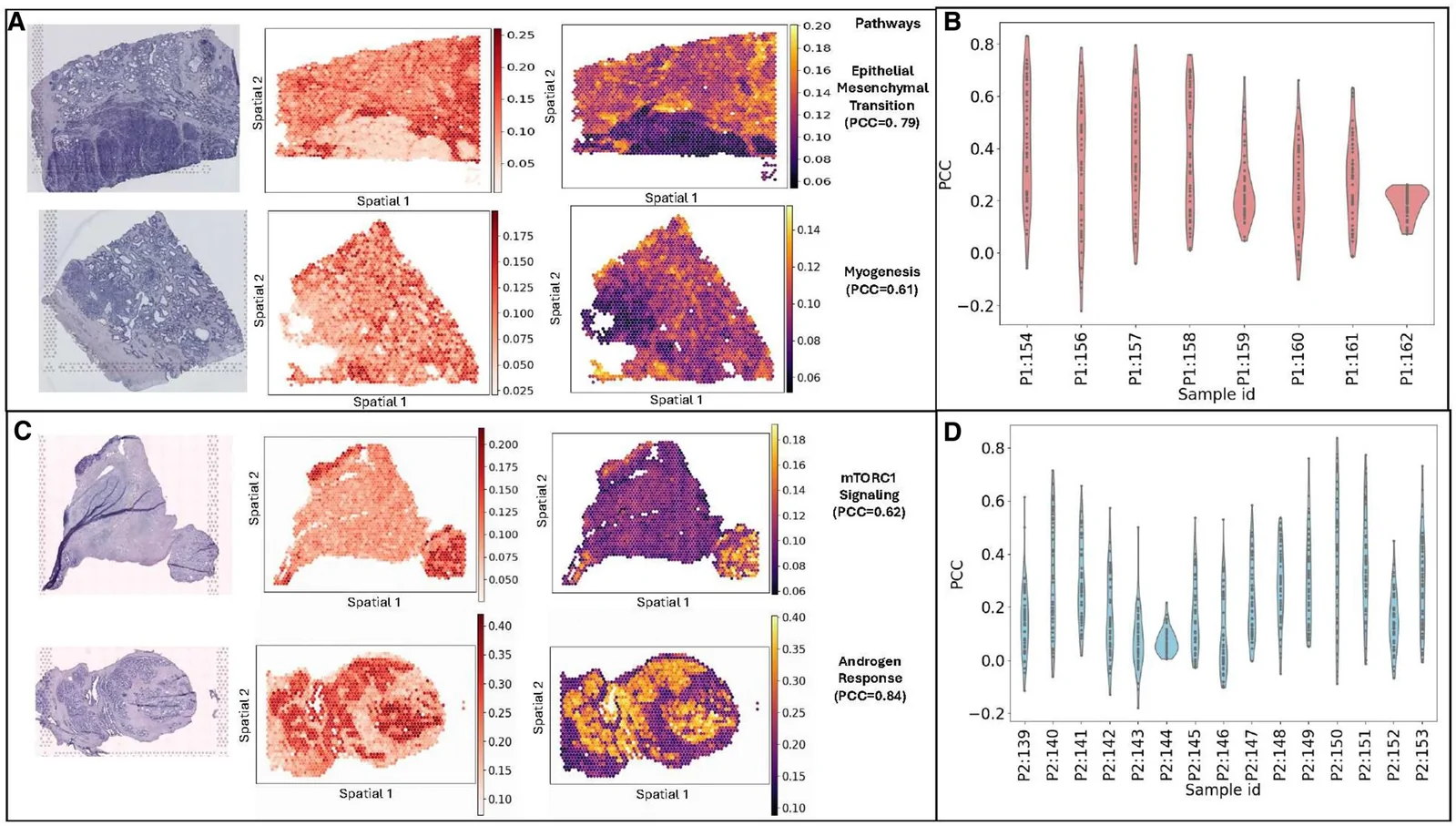
Evaluation under leave-one-patient-out settings. (A) Spatial visualization of top predicted pathways when DeepPathway model is evaluated using leave-one-patient-out cross validation on samples of Patient 1 (P1) only. (B) PCC distribution of each test sample of P1 when DeepPathway model is trained only on P2 samples. (C) Spatial visualization of selected top predicted pathways when DeepPathway model trained on samples of P1 only and evaluated on samples of P2. (D) PCC distribution of each test sample in P2 when DeepPathway model is trained only on samples of P1.

Here, DeepPathway model’s stage-1 (Bleep with Optimus) is evaluated using leave-one-patient-out approach. Figure S7 shows that for P1 and P2, most of the samples have achieved high PCC values when P1 and P2 are evaluated independently. For example, EMT pathway has achieved PCC values 0.69 and 0.81 for P1 (ID = 158) and P2 (ID = 150), respectively. Similarly, for stage-2 model (Fig. 4), a similar trend is observed, i.e. the model has performed reasonably well in predicting pathway expression on most of the test samples of P1 and P2. For example, EMT pathway is predicted with the PCC of 0.79 (ID = 156), whereas the Androgen Response is predicted with the PCC of 0.84 in P2 (ID = 150). This behavior shows that the model aligns well in a bimodal setting where the spatially localized patterns are present in both H&E and pathway expression. To support this further, we computed the inter-pathway correlation (PCC between each pathway with other pathway across all spots) using the predicted pathway expression obtained when DeepPathway is trained under leave-one-patient-out settings. Heatmap for the samples having ID = 157 from P1 and ID = 150 from P2, are shown in Figs. S9 and S10, respectively. Both results show that there is pathway expression level heterogeneity. For instance, for both ground truth and prediction (as shown in Fig. S10), p53 pathway and mTORC1 Signaling pathway are positively correlated. Furthermore, EMT and Myogenesis pathways are also positively correlated in both ground truth and predictions, co-exist in prostate cancers and contribute to tumour diversity, specifically, with respect to metastatic potential and differentiation state.

We also analysed the pathways predicted well within each tissue sample individually using both approaches. These pathways are selected from highly predicted pathways based on PCC scores. Spatial visualization of test samples evaluated from both mixed patients’ samples and leave-one-patient-out approaches, are shown in Figs. S11, S12, and S13, respectively. Additionally, we evaluated stage-1 and stage-2 of DeepPathway model on skin cancer dataset (Fig. S8), where 4 ST samples are evaluated using leave-one-out cross validation.

For the sake of evaluating DeepPathway on external and independent datasets, we used two prostate cancer ST samples (Mirzazadeh *et al.* 2023) from HEST-1k from a different study to predict the pathway expression. This dataset is processed independently, and we found out 20 out of 50 pathways present in the data. We predicted pathway expression from both models, i.e. using mixed patient samples model and the model trained on all samples from P1 and P2. We predicted 47 pathway expression and then selected those 20 pathways from predicted expression which are available in ground truth for spatial visualization and PCC computation (see Fig. S14). Additionally, we used small prostate cancer dataset as a part of external validation (see Fig. S15). Also, we additionally computed spatial statistics like Spearman correlation coefficient and moran’s I for all experimental settings (See Figs. S16–S20).

### 3.3 Pathway expression mapping in prostate cancer using TCGA whole slide images

**Figure 5:**
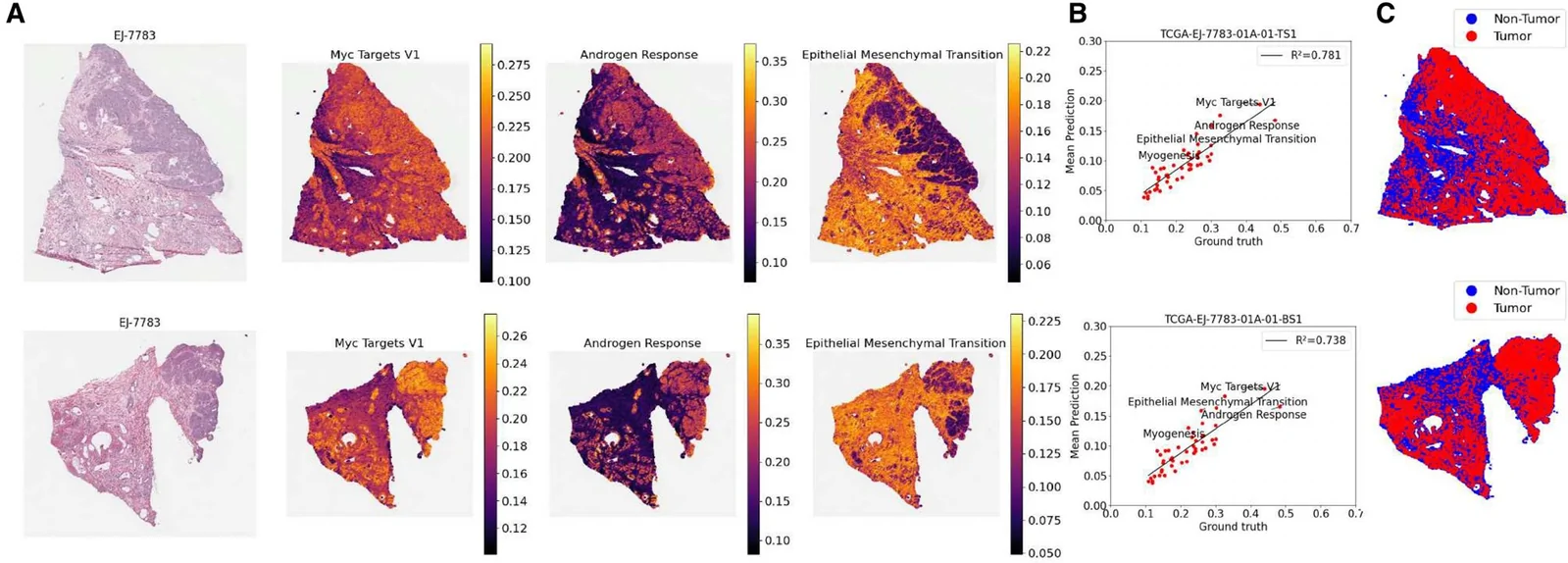
(A) H&E and spatial visualization obtained from the DeepPathway model for MyC Targets V1, Androgen Response, and Myogenesis on TCGA prostate tumor samples. (B) A scatter plot showing the quantitative results computed between bulk RNA-seq data and mean prediction. (C) Orthogonal validation with patch-wise tumor classification from the model trained on the TCGA prostate cancer dataset.

We extended the application of our model to predict pathway expression from prostate cancer dataset in the cancer genome atlas (TCGA); the most widely used public cancer data resource. We acquired WSIs of 8 patients which have both normal and prostate tumour tissue slides from TCGA data portal. We then used DeepPathway trained on combined samples of large prosate P1 and P2 to predict the patch-wise pathway expression using only WSIs. We selected three representative pathways, i.e, Myc Targets V1, Androgen Response, and EMT, for patch-wise visualisation. Myc is the major contributor in prostate cancer progression (Qiu *et al.* 2022). EMT drives tumour metastasis (Mittal 2018) while activation of androgen receptor signalling drives proliferation and survival of prostate cancer cells (Fujita and Nonomura 2019). Figure 5A shows the results of the predictions obtained for these three pathways on two TCGA tumour samples (row-wise). The results for remaining tumour patients tissue sections are shown in Fig. S21. Myc Targets V1 and Androgen Response show similar while EMT and Myc Targets V1 have inverse behaviour with respect to their expression values. To further assess the quality of pathway expression prediction on TCGA slides, we obtained the bulk rna-seq data and computed the ground truth pathway expression using Ucell. Then we averaged the patch-wise predictions to compare it with slide-level ground truth expression. Figure 5B shows the scatter plot along with $R^{2}$ value computed between ground truth and prediction for each sample. This shows that DeepPathway can effectively capture transcriptomic information from histopathology images. Finally, as an orthogonal validation, we used a pre-trained deep learning model (https://huggingface.co/kaczmarj/prostate-tumor-resnet34.tcga-prad) [used by WSInfer (Kaczmarzyk *et al.* 2024) in QuPath], trained on TCGA prostate cancer images to classify between tumour versus normal. Figure 5C shows the patch-wise classification result, which confirms that DeepPathway have predicted the pathways like MyC Targets V1 or Androgen Response with high expression values in active tumour regions. Additionally, results for normal tissue sections are shown in Fig. S22.

### 3.4 Prediction of hypoxia signatures from H&Es of brain tumour patient samples

Hypoxia is one of the hallmarks of cancer and it is caused by the unbalanced oxygen availability due to insufficient blood supply. It is also known to promote tumour progression and metastatic spread (Muz *et al.* 2015, Chen *et al.* 2023, Feldman 2024). Hypoxia can be traced in tissues using pimonidazole (PIMO) which binds selectively thiol-containing proteins or forms metabolites after reduction of its nitro group macromolecules when oxygen concentration falls below 10 mmHg (Kizaka-Kondoh and Konse-Nagasawa 2009).

**Figure 6:**
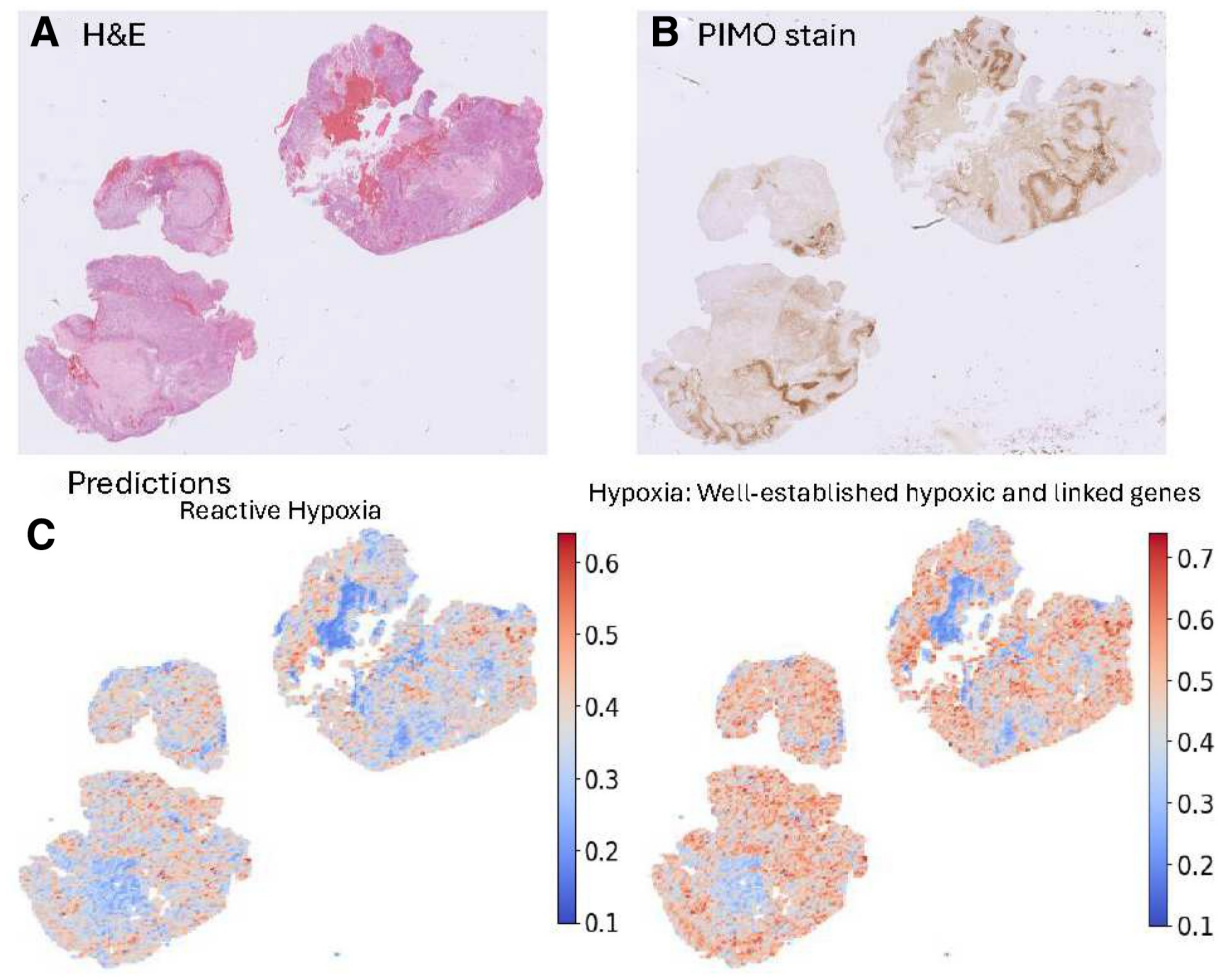
(A) Visualization of H&E image, (B) PIMO staining validation, and (C) Predicted Reactive Hypoxia pathway and a custom hypoxia geneset comprised of well-established hypoxic genes along with their linked genes from H&Es of brain tumour using DeepPathway.

Here we applied DeepPathway to the samples from patients with GBM who received PIMO as part of a clinical trial (Waqar *et al.* 2023). Consecutive sections were stained for H&E and PIMO as ground truth and analysed with DeepPathway. We selected five brain tumour specific pathway definitions from Ravi *et al.* (2022), which include (i) Reactive Hypoxia, (ii) Radial Glia, (iii) Reactive immune, (iv) Regional Neural Progenitor Cell (NPC), and (v) Regional Oligodendrocyte Precursor cell (OPC). Additional two pathway definitions we included are the subset of Reactive hypoxia definition, where one definition include 20 well established hypoxia specific genes while other subset include 41 genes (sum of 20 well established and 21 genes associated with hypoxic genes), curated locally. We trained DeepPathway model on five GBM samples (Ravi *et al.* 2022). We used in-house brain-tumour patients H&Es to predict the hypoxia pathway expression. Results for GBM patient having hypoxia clearly marked with PIMO stain is shown in Fig. 6 which shows that DeepPathway is able to mark certain hypoxic areas using only H&Es which is typically much harder task for the pathologist (See Fig. S23 for other patients). This approach provided a robust validation of DeepPathway and of its translational potential, providing a good visual concordance with the PIMO stain despite being trained on limited and low-quality ST brain tumour specific data. Indeed this will need further validation for model generalisation on routine H&Es going forward for any translational applications.

## 4 Discussion and conclusion

Here, we proposed DeepPathway, a novel approach for the prediction of pathway expression from H&E images. Trained on ST data—this approach successfully links visual patterns in histological images to functional biological processes. H&E images are routinely available but their connection to functional biology remains largely uninterpreted. Existing AI models which predict expression of individual genes from histology fail to capture the cooperative nature of genes acting in pathways and can be noisy and biologically un-intuitive. DeepPathway addresses these gaps by linking tissue morphology and functional biology, aiming to extract a rich, pathway-level understanding of a tumour’s biology directly from a standard, low-cost image.

Our method builds on existing work for the prediction of gene expression from H&E images and we establish the utility of pathway prediction directly from H&Es, as compared to individual gene expression prediction followed by pathway quantification. For both gene and pathway expression prediction tasks, quality of images and their pre-processing could have significant impact on the model development and performance. DL-based expression prediction models mainly use spot-level H&E image patches, which are cropped around the center of the spots. However, in ST data, tissue sections may have variable image resolution due to the technical differences between experiments or variable spot diameters even using the same technology like 10X Visium. This leads to H&E patch sizes with inconsistent morphological features. Additionally, in clinical settings, tissue H&Es without ST data can be stored in standard imaging formats like portable network graphics (PNG), where the image resolution in microns per pixel (MPP) is unavailable. To achieve the constant-sized image patch which is not affected by the spot diameter or the type of image, we have used TIA toolbox which provides patches of same size (say 224×224) with any given MPP resolution. Additionally, large-scale ST datasets, e.g. HEST-1k provides normalized and transformed WSIs in a pyramidal objects (TIFF) along with the metadata. However, in future, a multi-resolution DL-based framework for leveraging different magnifications will be needed to achieve more generalizable results.

Besides images, batch correction of multi-sample gene expression data might be needed before training the DL models for gene or pathway expression prediction. Methods like Harmony (Korsunsky *et al.* 2019) or single-cell variational inference (Lopez *et al.* 2018) are used to remove batch effects, could result into negative gene expression values. In our work, we have not applied any batch correction for the sake of obtaining positive valued gene expression prediction that are used to obtain reconstructed pathway expression.

We used UCell to quantify pathway expression/activity score from measured gene expression data. It computes pathway scores based on within-spot gene rankings, with the Rmax parameter limiting the contribution of genes ranked beyond a specified threshold. As the same scoring procedure and parameter settings were applied uniformly across all samples and experimental conditions, any impact of this heuristic is expected to be consistent across comparisons. Moreover, by relying on relative gene ranks rather than absolute expression, UCell is relatively robust to library-size and global scaling differences. However, UCell-derived pathway scores may still be influenced by data sparsity, and sample-specific preprocessing choices, e.g. Rmax value.

Recently, H&E foundation models are being widely applied to extract rich histology features as they are trained on enormous amount of WSIs. We have used the first version of optimus foundation model, i.e. *H-optimus-0*, however, there are other foundation models (Chen *et al.* 2024, Vorontsov *et al.* 2024) along with *H-optimus-1* (https://huggingface.co/bioptimus/H-optimus-0-1) (trained on histology images sampled from over 1 million slides of more than $8,00,000$ patients) can be used as a future work, potentially leading to development of a pathway expression foundation model that can be used for multiple tissues.

Furthermore, we show the utility of our model on TCGA data where there are only H&E images available besides bulk RNA data. Our method can show spatially localized expression of different pathways using H&Es, differentiating expression on tumour vs normal tissue slides for same patients. Although we quantitatively compared mean DeepPathway predictions with slide-level RNA data but this is indeed limited comparison as spatial heterogeneity is not preserved.

We focused on GBM samples as a paradigm of hypoxic tumour, showing that DeepPathway can identify hypoxia in H&E images. Noteworthy, hypoxia can only be identified using the stain for HIF-1α as a surrogate biomarker of low oxygen concentrations. This was an excellent opportunity for validation and potential clinical utility of our method, as we have access to these precious human brain tumour samples which were stained with PIMO as a ground truth for hypoxia. Our method makes good visual concordance with the PIMO stain despite being trained on limited and low-quality ST brain tumour data. These analyses can be further extended and validated to make the case for potential clinical applications for prediction of hypoxia in many cancer tissues.

Lastly, although DeepPathway was evaluated using multiple publicly available ST datasets spanning multiple patients and tissue sections as well as it employs uses standard regularisation strategies but larger cohorts are always desirable for training and validation to mitigate overfitting. Hence, evaluation on larger multi-centre cohorts will be important to further assess robustness, and generalisability across patients in future.

Overall, to the best of our knowledge, this is the first approach to predict pathways from H&E images, and we believe with more training data, better quality images, refined pathway definitions, it will lead to further improvement and wider translational impact. In future, we will extend DeepPathway for high-definition ST platforms such as Visium HD, which offers near-cellular resolution as future work.

## Supplementary Material

btag643_Supplementary_Data

## Data Availability

All spatial transcriptomics datasets used in this study are publically available. The brain tumour dataset is available on Figshare (https://doi.org/10.48420/33187689) with ethics approval RAS: 244538 REC reference: 19NE0186.
