# Supplementary material for: DeepPathway: predicting pathway expression from histopathology images
Source: Bioinformatics. 2026 Aug 26;42(9):btag643. doi: 10.1093/bioinformatics/btag643 (PMC13585171; doi:10.1093/bioinformatics/btag643)
Supplement: Supplementary file 1 [file btag643_supplementary_data.pdf]

Supplementary Material

DeepPathway: Predicting Pathway Expression from Histopathology Images

Muhammad Ahtazaz Ahsan[1], Karen Piper Hanley[1], Martin Fergie[1], Claire O'leary[1], Gerben Borst[1], Federico Roncaroli[1], Fayyaz Minhas[2], Magnus Ratrray[1], Mudassar Iqbal[1*], and Syed Murutza Baker[1,3*]

[1]Faculty of Biology, Medicine and Health, The University of Manchester, UK
[2]Department of Computer Science, University of Warwick, Coventry, UK
[3]Advanced Bioinformatics and Computational, University of Manchester, UK

Sr. No.	Dataset	Number of Samples	Total Number of Spots	Image Resolution (MPP)
1	Small prostate cancer	5	19,245	0.61
2	Large prostate cancer	8 (Patient 1)<br>15 (Patient 2)	18,965 (Patient 1)<br>34,845 (Patient 2)	0.57, 0.68, 0.17 (Patient 1)<br>0.34 (Patient 2)
3	Skin cancer	4	8839	0.17
4	Brain tumour (Glioblastoma)	5	17425	0.46

Supplementary Table I: Statistics for the datasets used in this study.

Hyperparameter	Value
Training and validation split ratio (%)	80,20
Embedding dimension (ResNet50)	2048
Embedding dimension (ResNet18)	512
Embedding dimension (H-optimus)	1536
Embedding dimension (Positional Encodings)	256
L1 penality control parameter (λ)	0.0001
Patience level for preventing early stopping	5
Learning rate	0.001
Evaluation Metrics	Pearson Correlation Coefficient (PCC)<br>Spearman Correlation Coefficient<br>Moran's I

Supplementary Table II: Hyparameters and their values used in DeepPathway.

* Joint corresponding authors

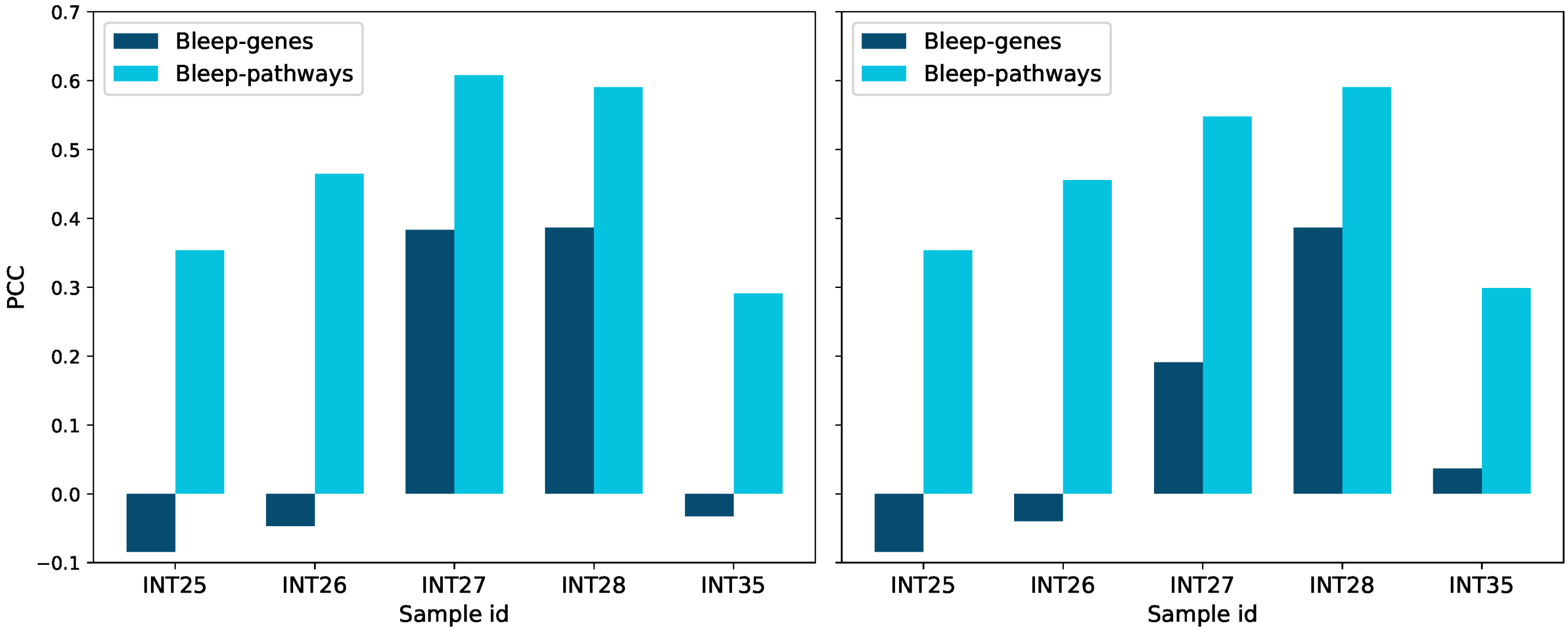

Supplementary Fig. 1: Average PCC of top 5 highly expressed **(left)** and highly variable **(right)** pathways obtained from baseline method, i.e., BLEEP-pathways and BLEEP-genes, on Small prostate data.

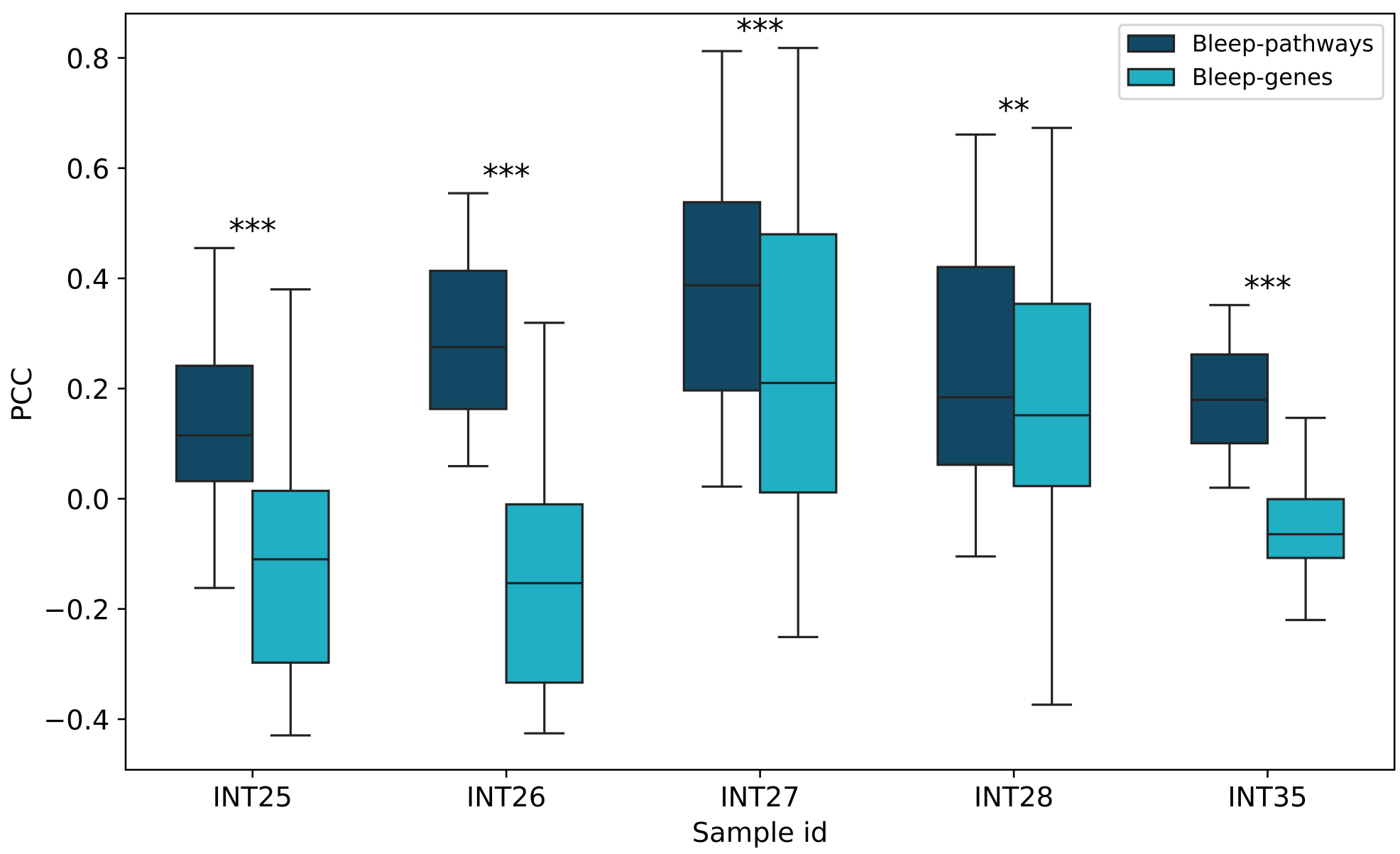

Supplementary Fig. 2: PCC distribution of Bleep-pathways model when trained with ResNet18 as an image encoder for small prostate cancer dataset.

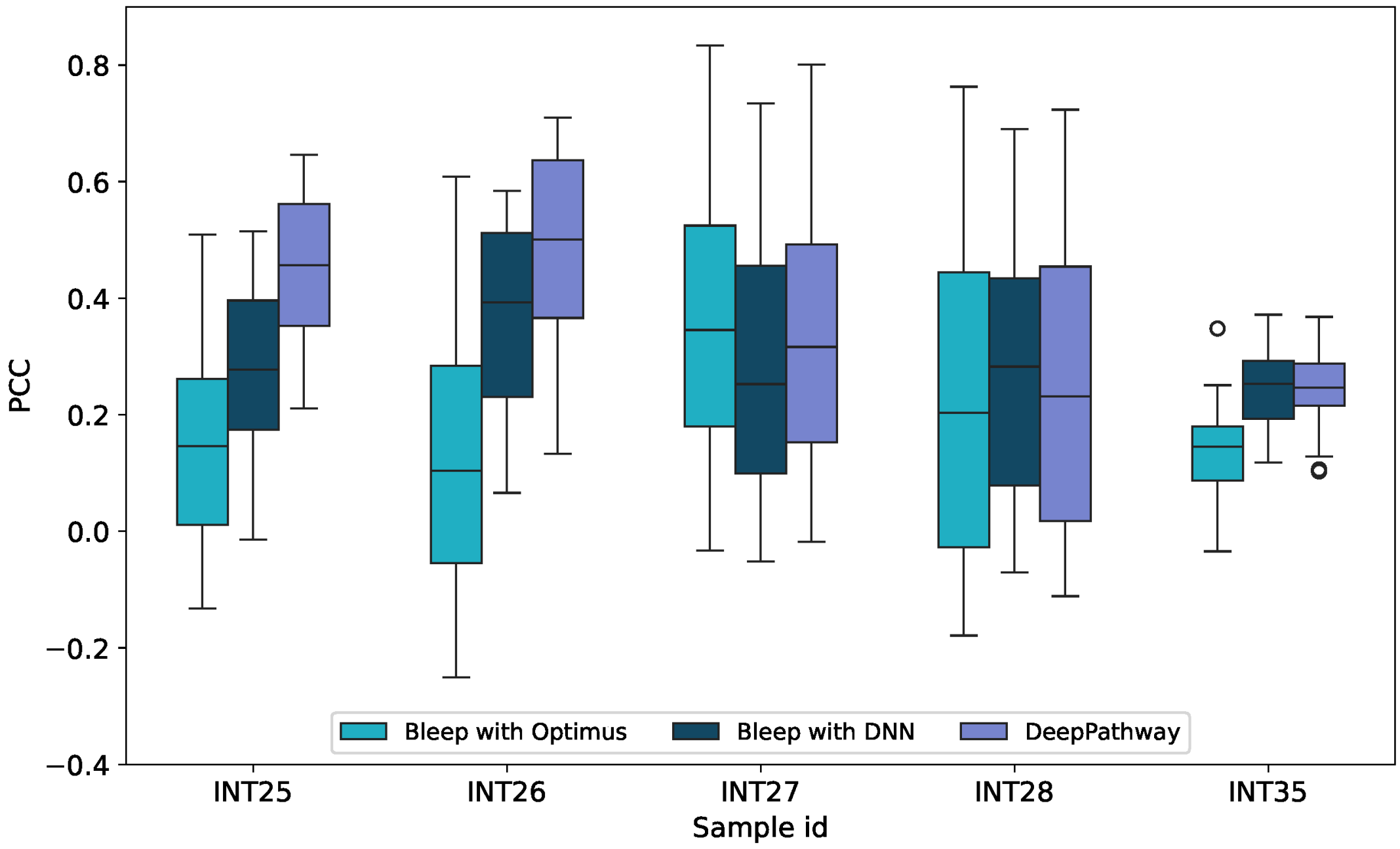

Supplementary Fig. 3: Validation study of DeepPathway (BLEEP with Optimus and DNN) by removing different components of the model on small prostate cancer data where image encoder is ResNet18 for every model.

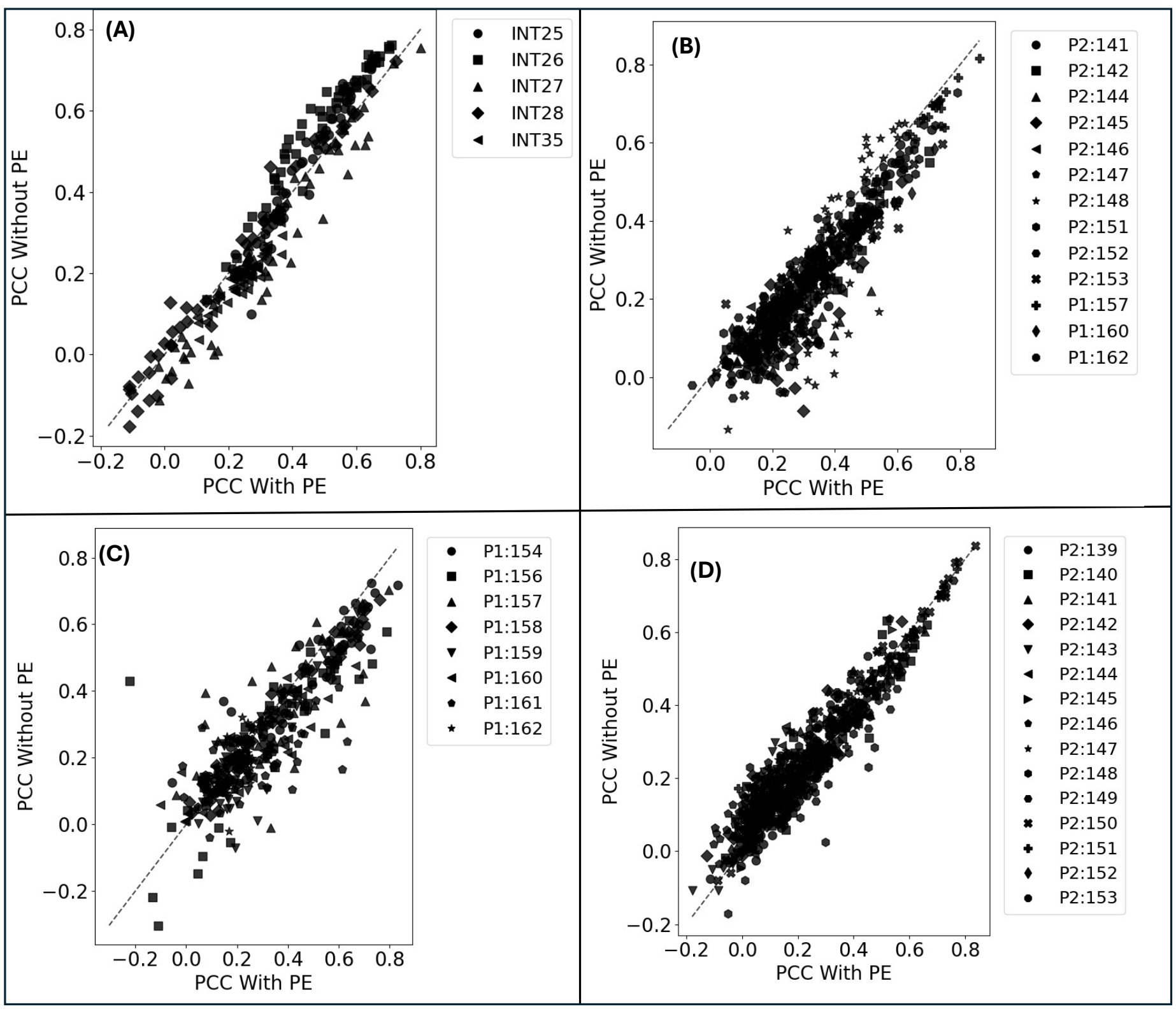

Supplementary Fig. 4: Ablation study of DeepPathway on different datasets with and without positional encodings (PE). **(A)** Small prostate cancer dataset. **(B)** mixed patient sample approach **(C)** Patient 1 is evaluated on trained model of Patient 2 samples. **(D)** Patient 2 is evaluated on trained model of Patient 1 samples.

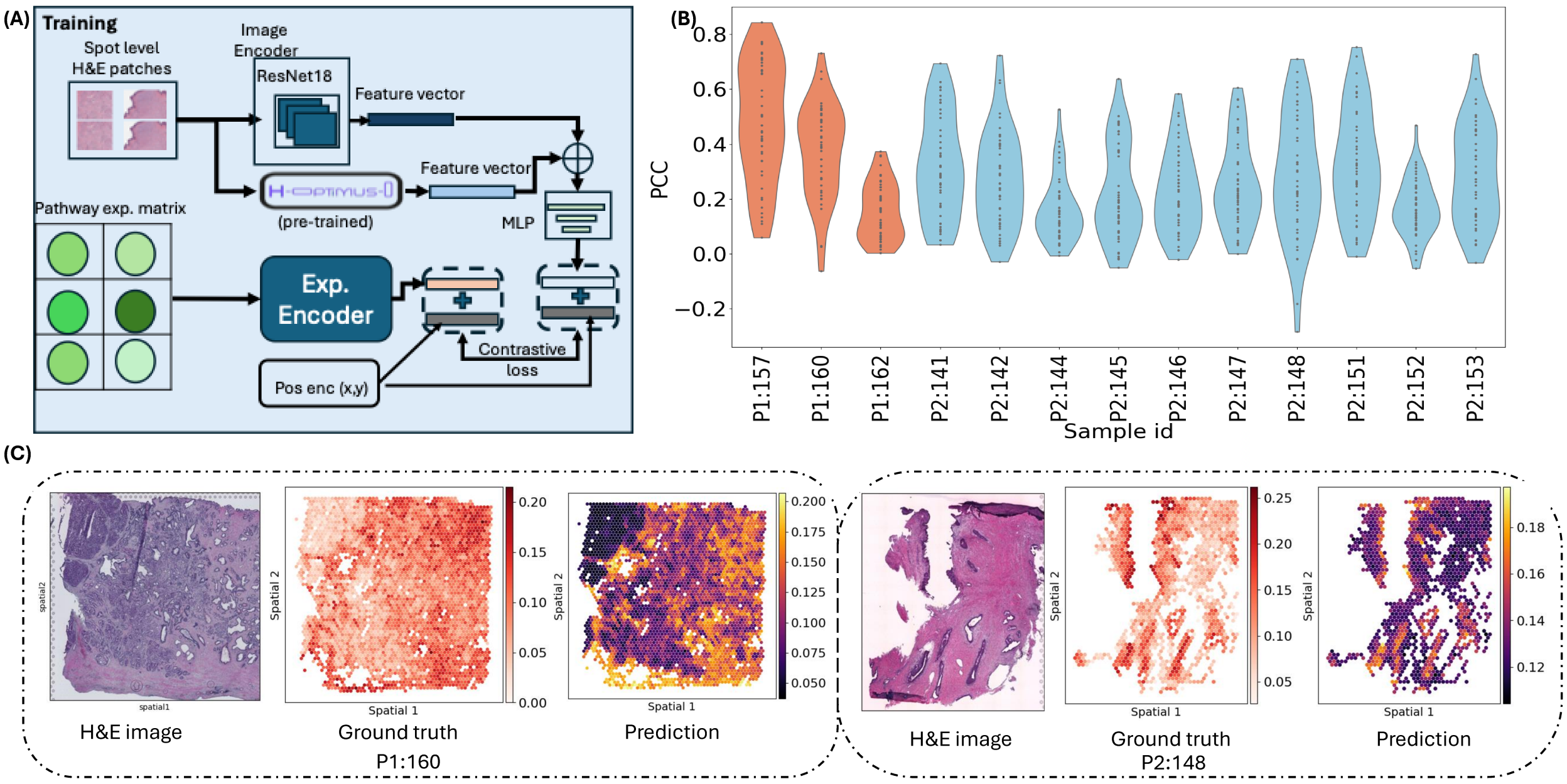

Supplementary Fig. 5: **(A)** Integration of H-Optimus-0 H&E foundation model with BLEEP (Stage-1 of DeepPathway model) to predict pathway expression from mixed samples of Patient 1 and Patient 2 of large prostate cohort. A combined 2048-dimensional feature vector (1536 from non-trainable H-Optimus-0 foundation model and 512-dimensional from trainable ResNet18) is inputted to BLEEP for model training and validation. **(B)** PCC distribution plot of test samples taken from Patient 1 and Patient 2 **(C)** test H&E image and spatial visualization of the Epithelial Mesenchymal Transition (P1:160) and p53 Pathway (P2:148) ground truth pathway expression—obtained from UCell—alongside the predicted pathway expression. The predicted expression achieved a PCC values of 0.73 and 0.71, respectively.

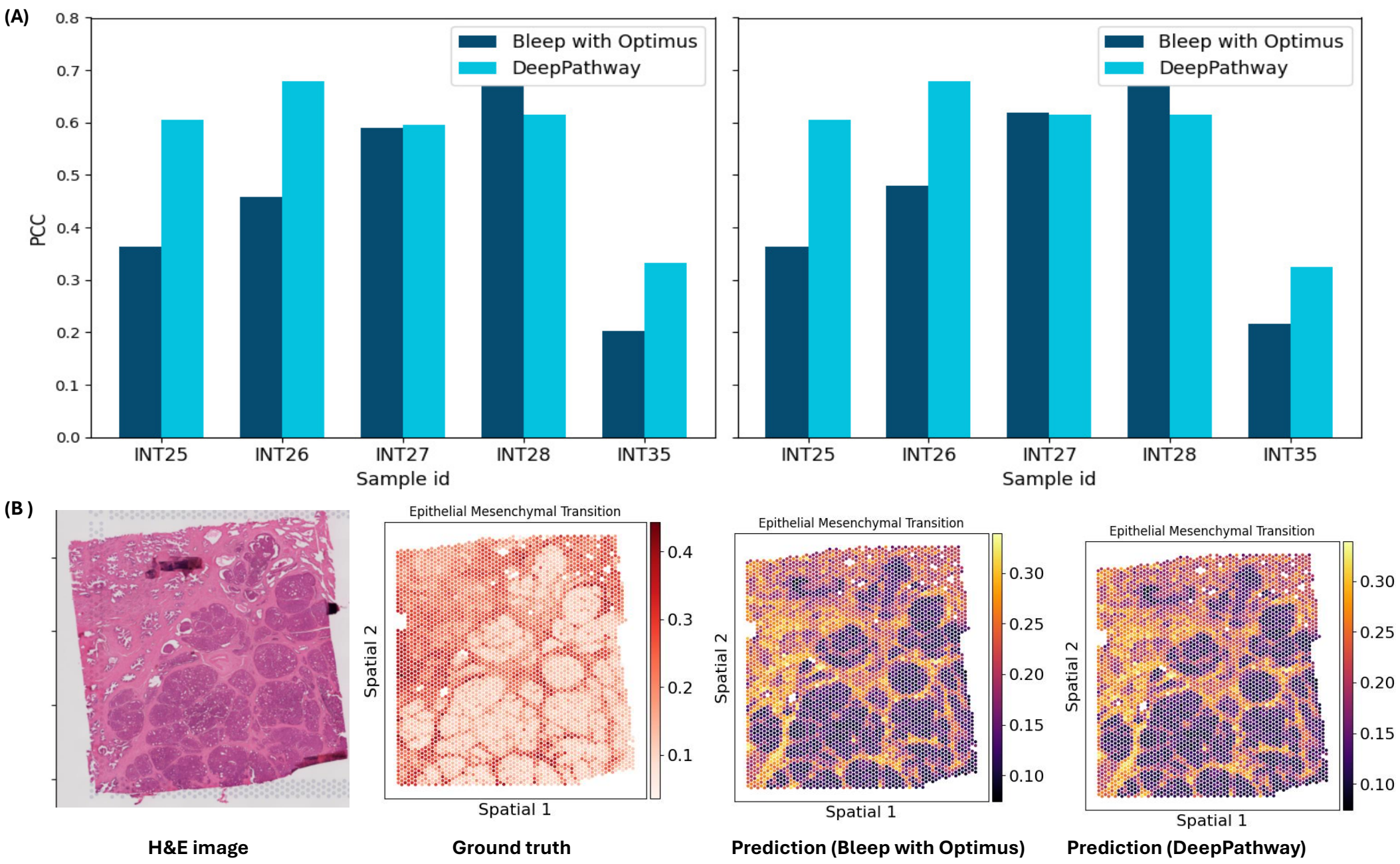

Supplementary Fig. 6: **(A)** Average PCC of top 5 highly expressed **(left)** and highly variable **(right)** pathways obtained from Bleep with Optimus (Stage-1) and DeepPathway. **(B)** H&E and spatial visualization of the ground truth (obtained from the UCell) and predictions obtained from both models. The predicted expression achieved the PCC of 0.83 and 0.80 for the Epithelial Mesenchymal Transition pathway (sample ID=INT27).

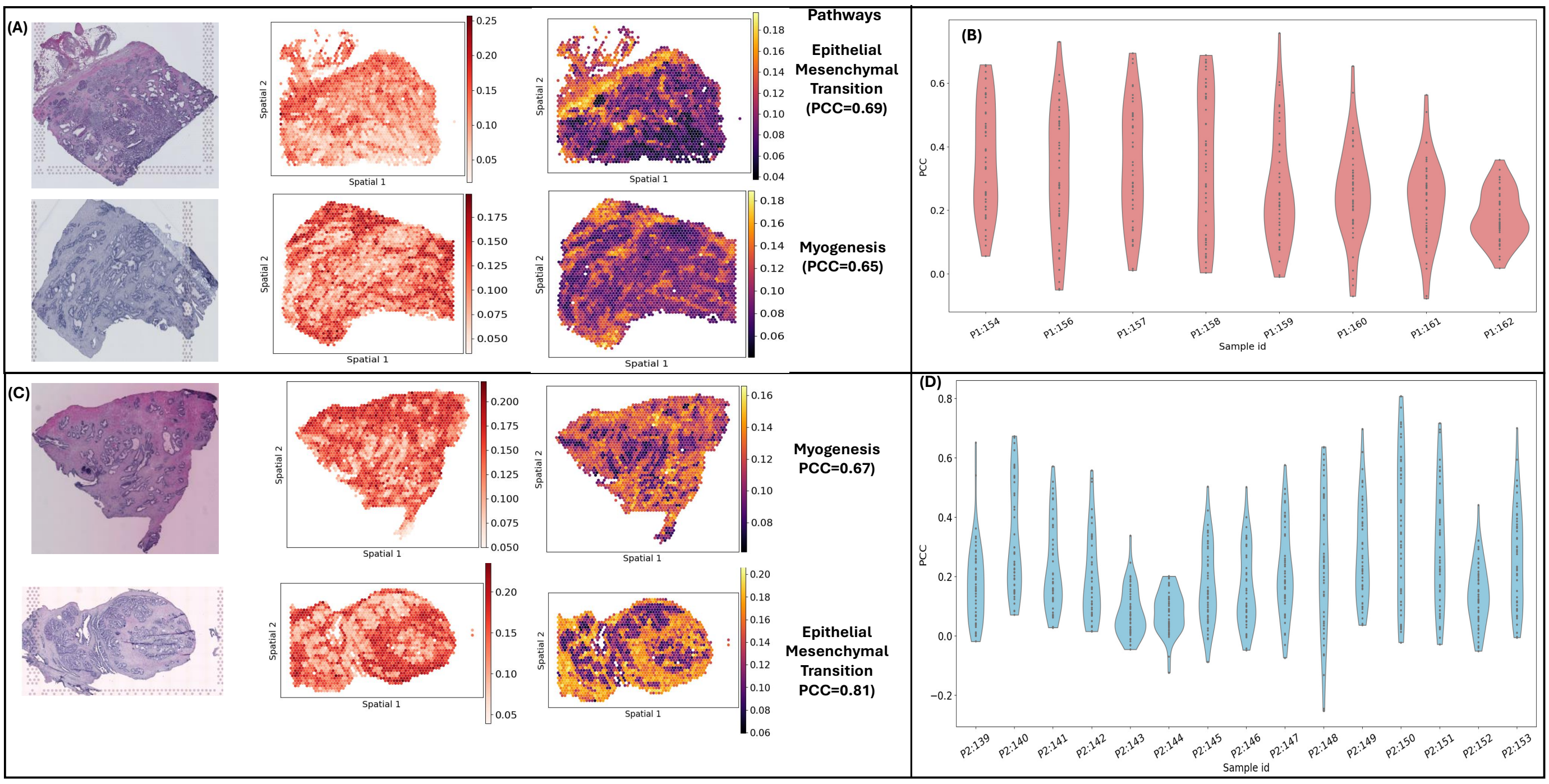

Supplementary Fig. 7: Patient-wise evaluation of Bleep with Optimus (Stage-1 of DeepPathway model) using leave-one-patient-out cross validation. **(A)** H&E along with spatial visualization of ground truth and prediction obtained for best predicted pathways from Patient 1 samples (Ids=158,154). **(B)** PCC distribution of all pathways for all test samples of Patient 1. **(C)** H&E along with spatial visualization of ground truth and prediction obtained for best predicted pathways from Patient 2 samples (Ids=140,150). **(D)** PCC distribution of all pathways for all test samples of Patient 2.

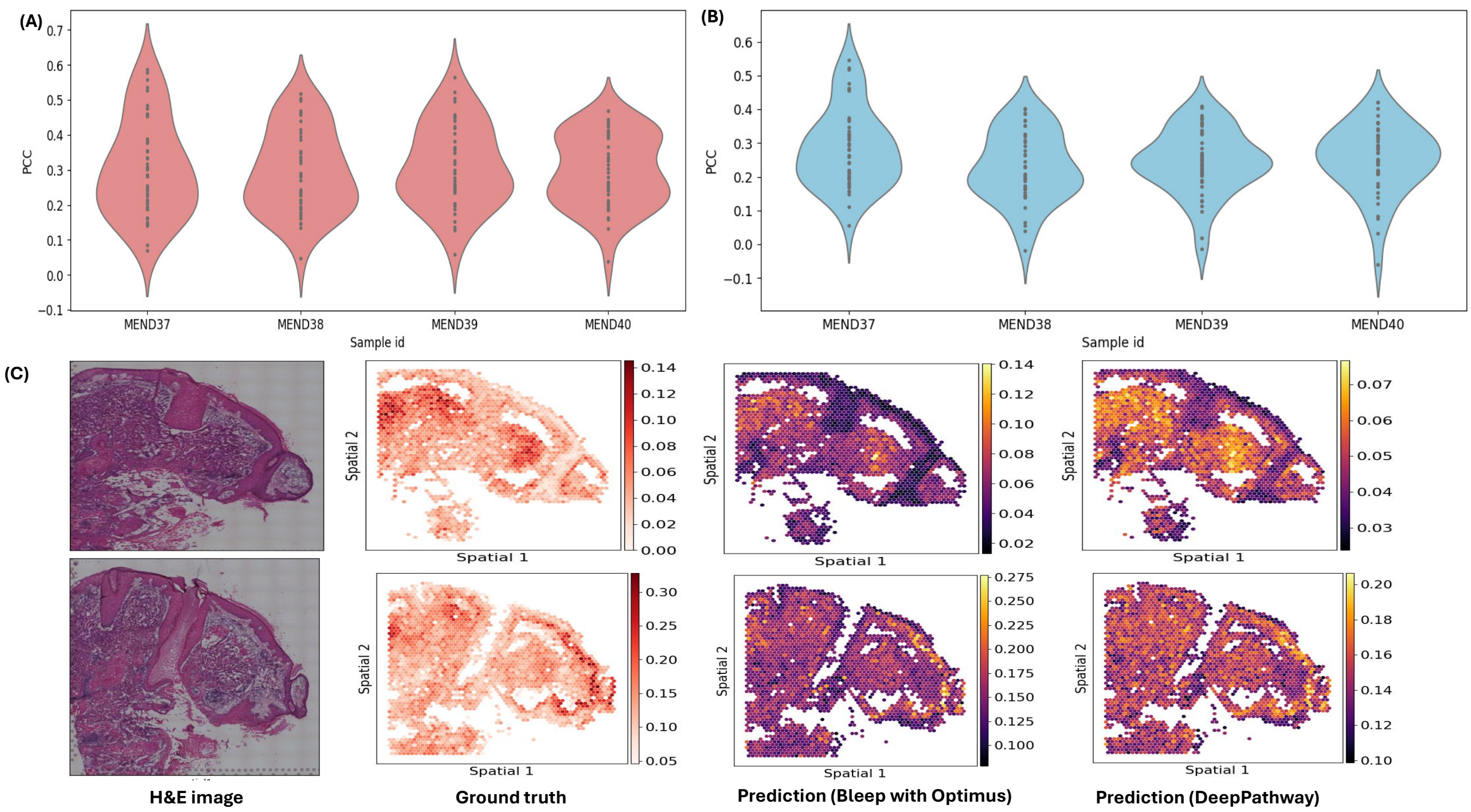

Supplementary Fig. 8: **(A)** PCC distribution of each sample in leave-one-out cross validation on skin cancer data using Stage-1 of DeepPathway (BLEEP with *H-Optimus-0* model). **(B)** PCC distribution of each sample in leave-one-out cross validation on skin cancer data using DeepPathway **(C)** H&E and spatial visualization of the ground truth (obtained from the UCell) and predictions obtained from both models. The predicted expression achieved the PCC of 0.59 and 0.52 for stage-1 and stag-2, respectively, for E2F Targets pathway (top row, sample ID=MEND37), while achieved the PCC of 0.52 and 0.41, for Myc Targets V1 pathway (bottom row, sample ID=MEND39).

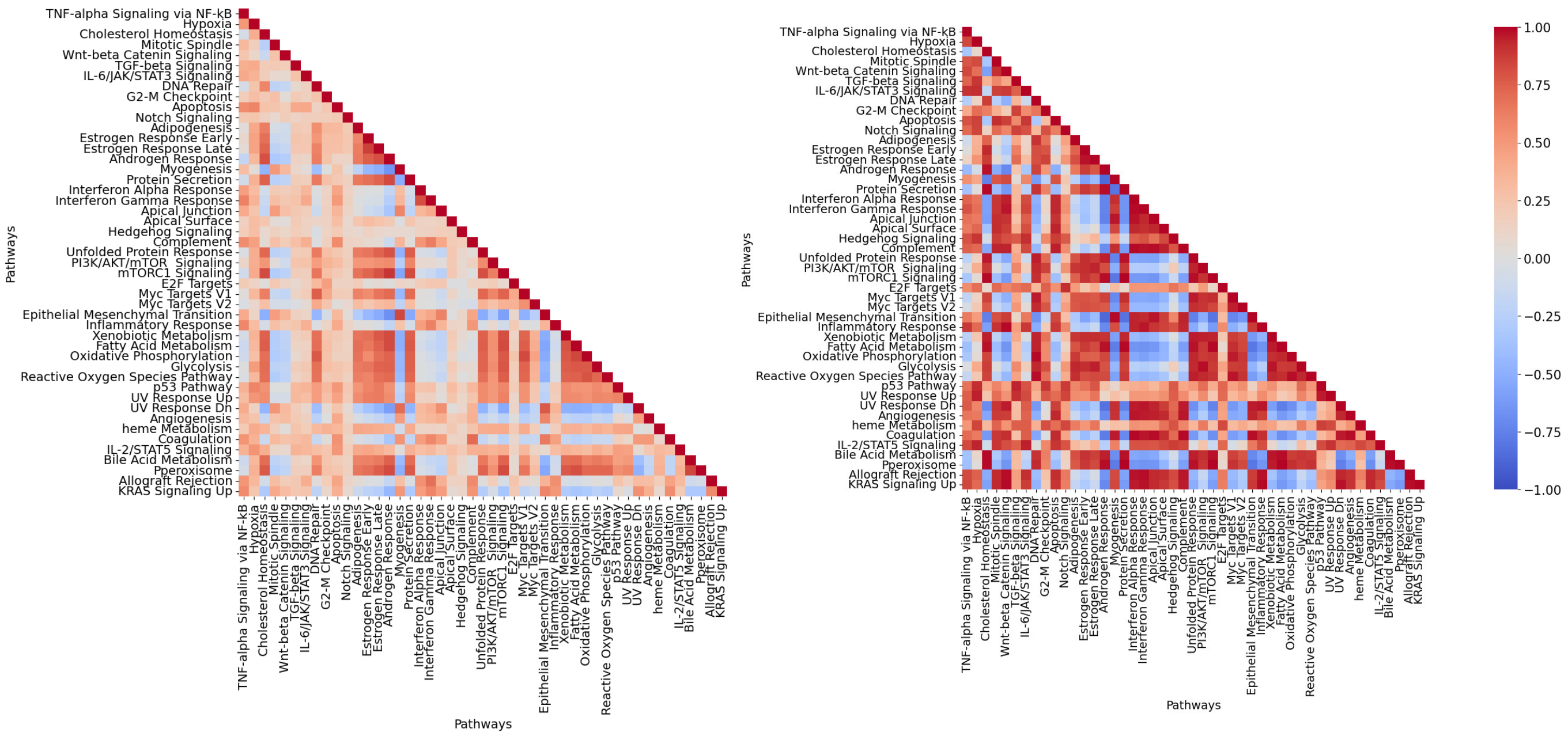

Supplementary Fig. 9: Intra-correlation computed between ground truth and predicted pathway expression (from DeepPathway model trained on Patient 2 samples only) for sample id=157, computed using 47 pathway definitions obtained from MSigDB hallmark on large prostate cancer data.

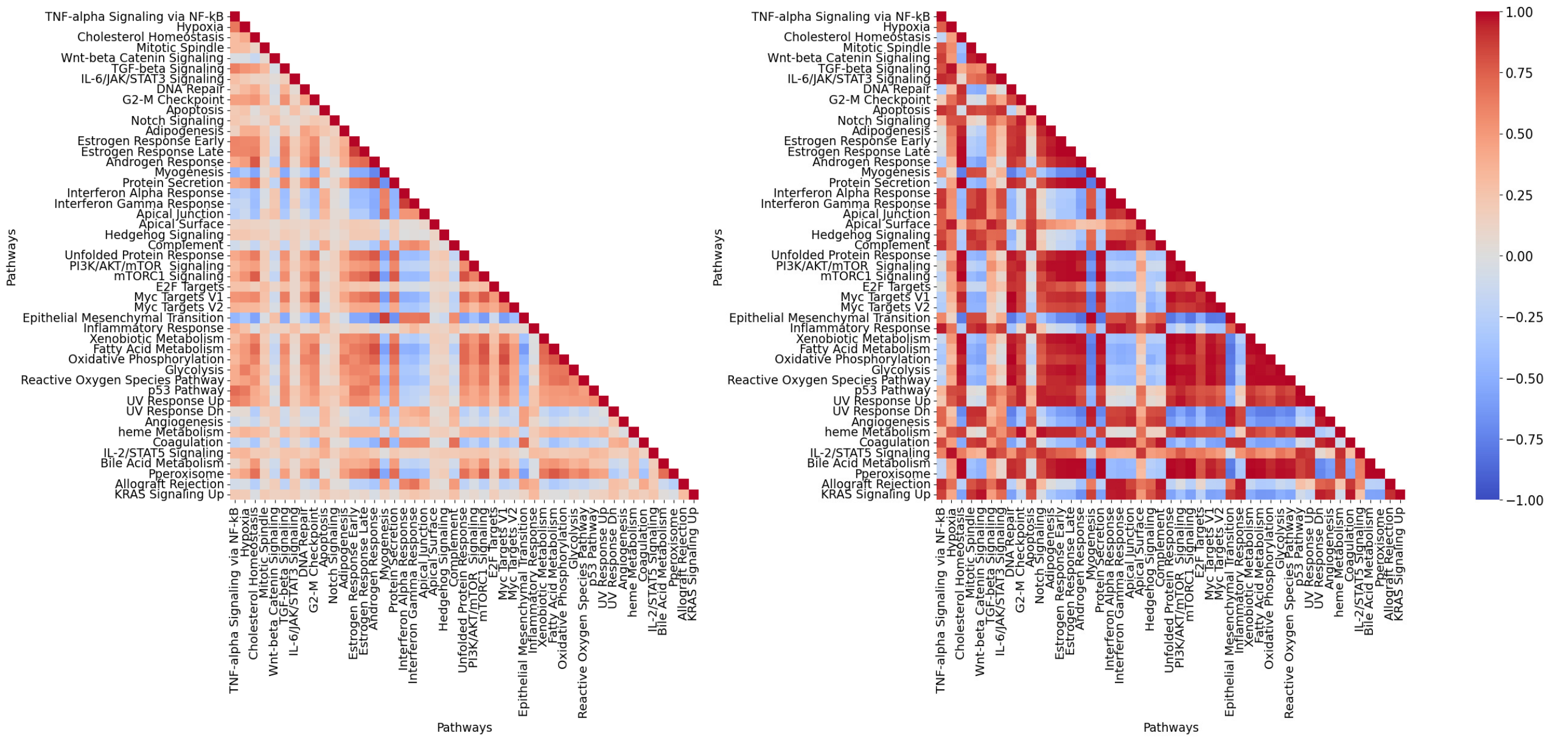

Supplementary Fig. 10: Intra-correlation computed between ground truth and predicted pathway expression (from DeepPathway model trained on Patient 1 samples only) for sample id=150, computed using 47 pathway definitions obtained from MSigDB hallmark on large prostate cancer data.

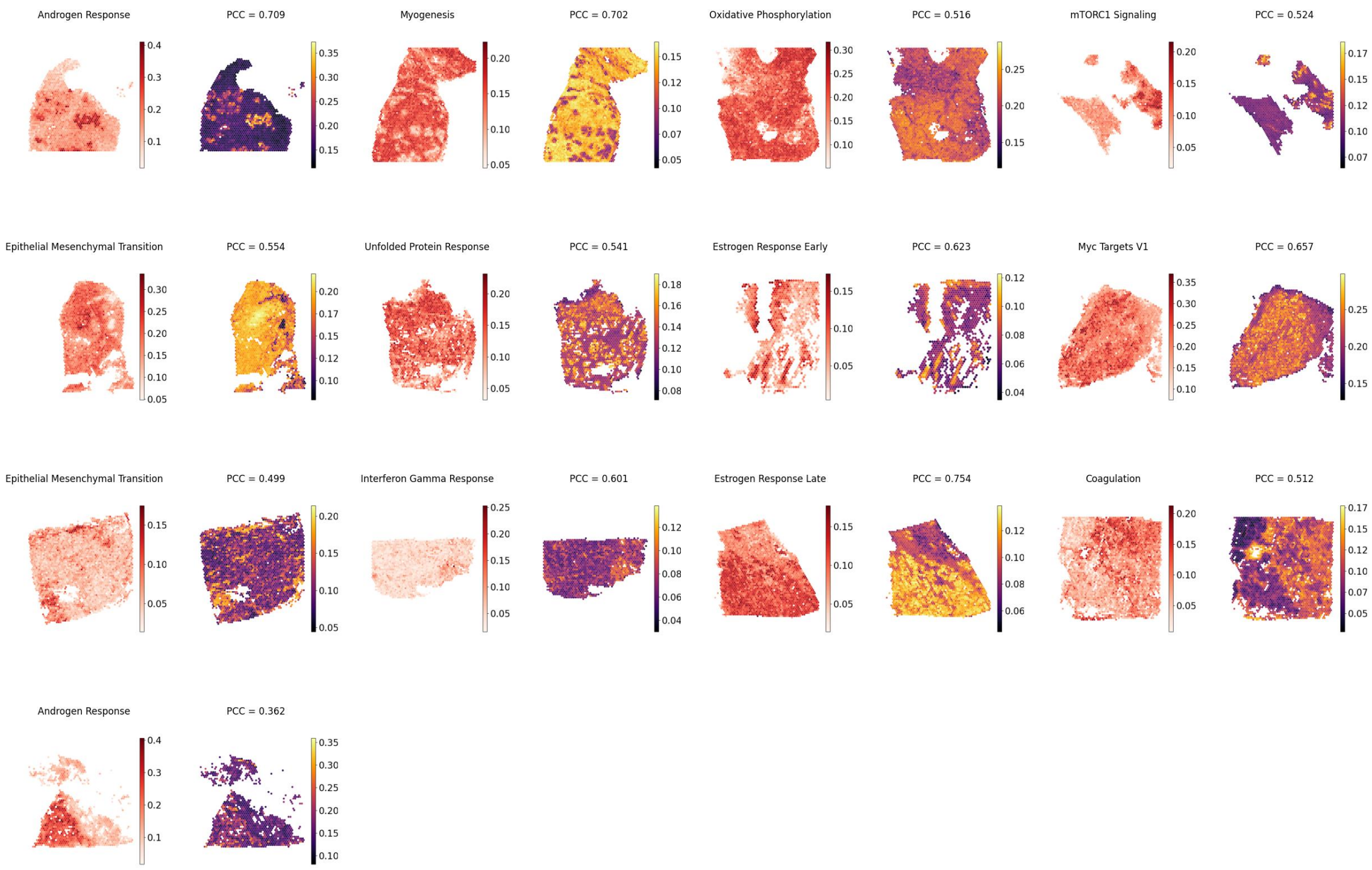

Supplementary Fig. 11: Spatial visualization of pathways predicted well within all slides from DeepPathway model using mixed patients' samples approach.

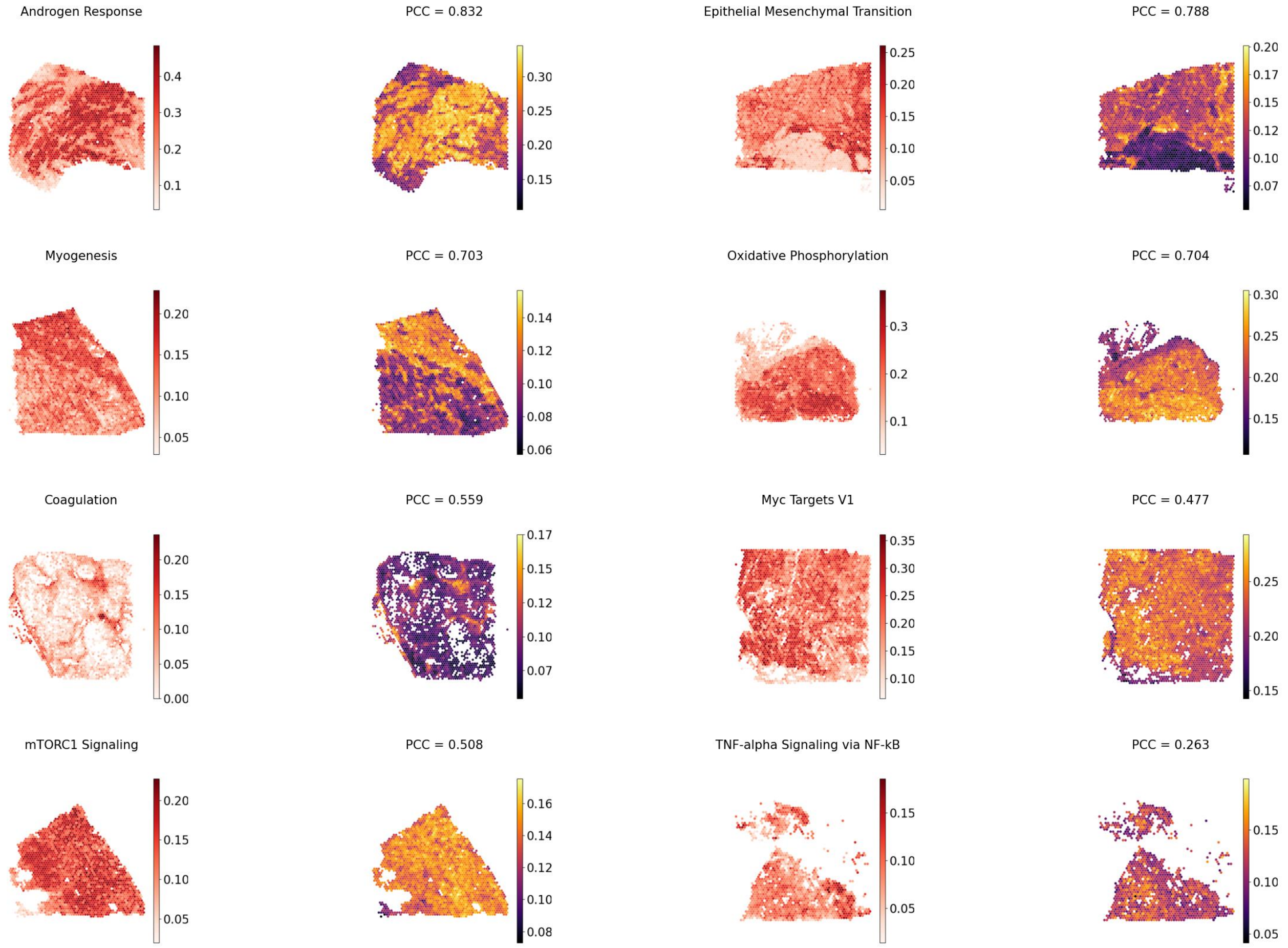

Supplementary Fig. 12: Spatial visualization of pathways predicted well within all slides from DeepPathway model applied on all samples of test Patient 1 (model trained on P2 samples only).

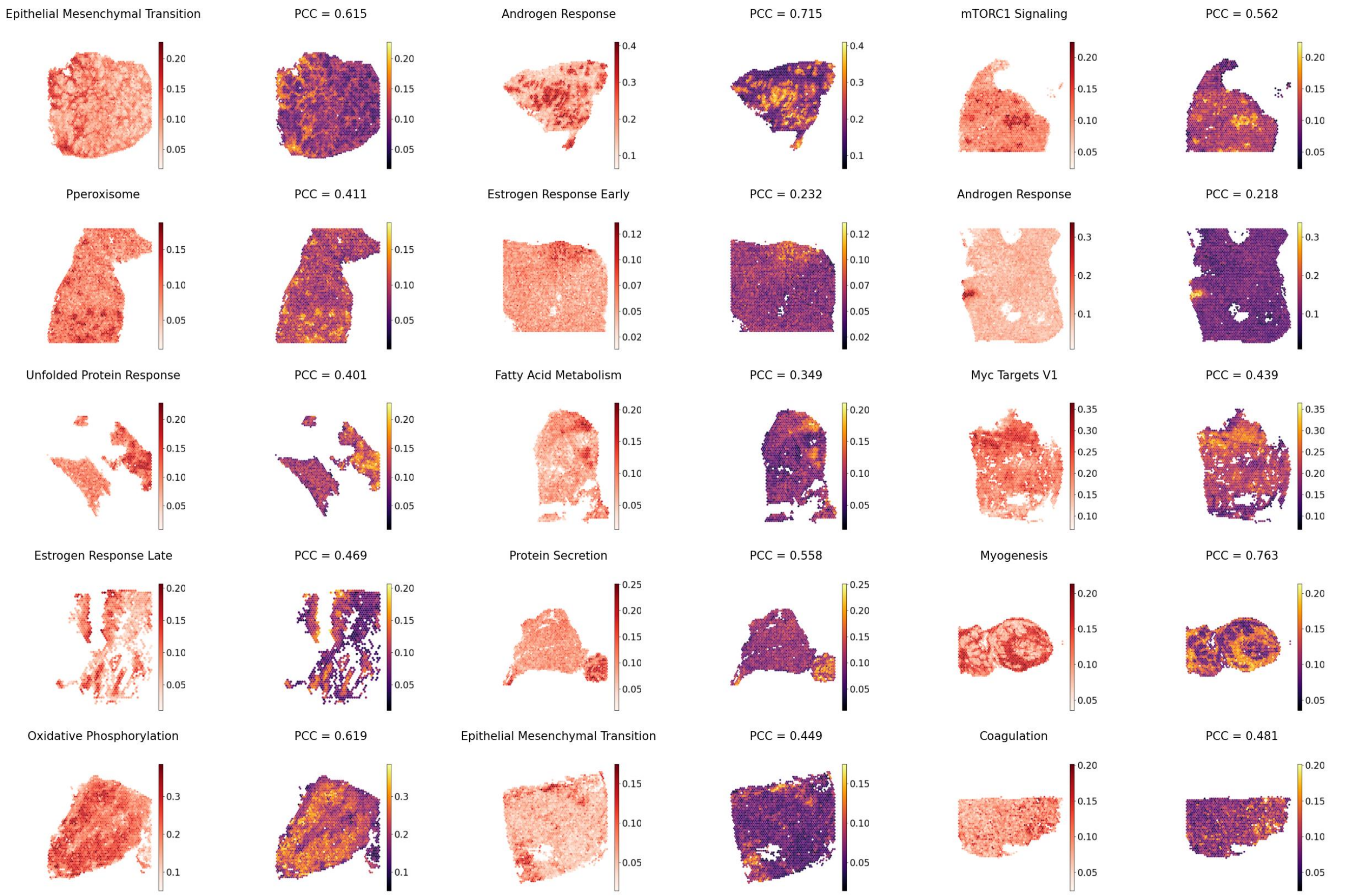

Supplementary Fig. 13: Spatial visualization of pathways predicted well within all slides from DeepPathway applied on all samples of test Patient 2 only (model trained on P1 samples only).

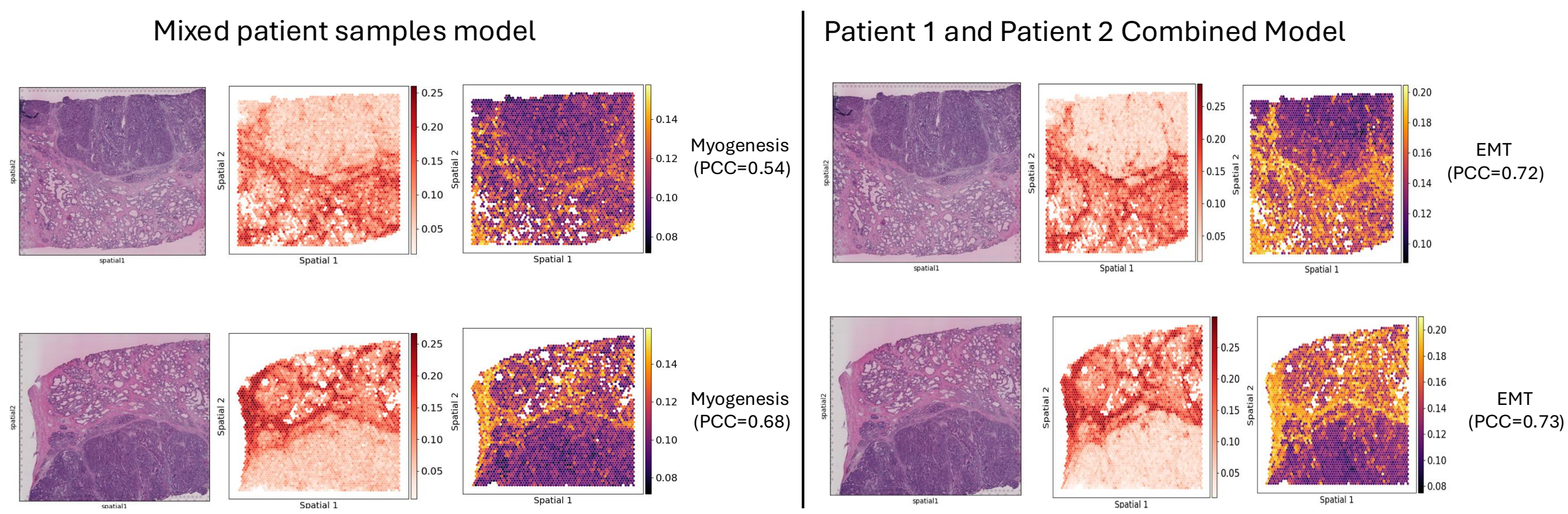

Supplementary Fig. 14: Evaluation of DeepPathway model using two samples, i.e., MEND61 (top-row) adn MEND62 (bottom-row) from another prostate cancer study (taken from HEST-1k) data as an independent data cohort where the model is trained (i) using mixed patient samples of P1 and P2 **(left)** and (ii) using model trained only on samples of P2 ((**right**)) for showing its robustness and generalizability.

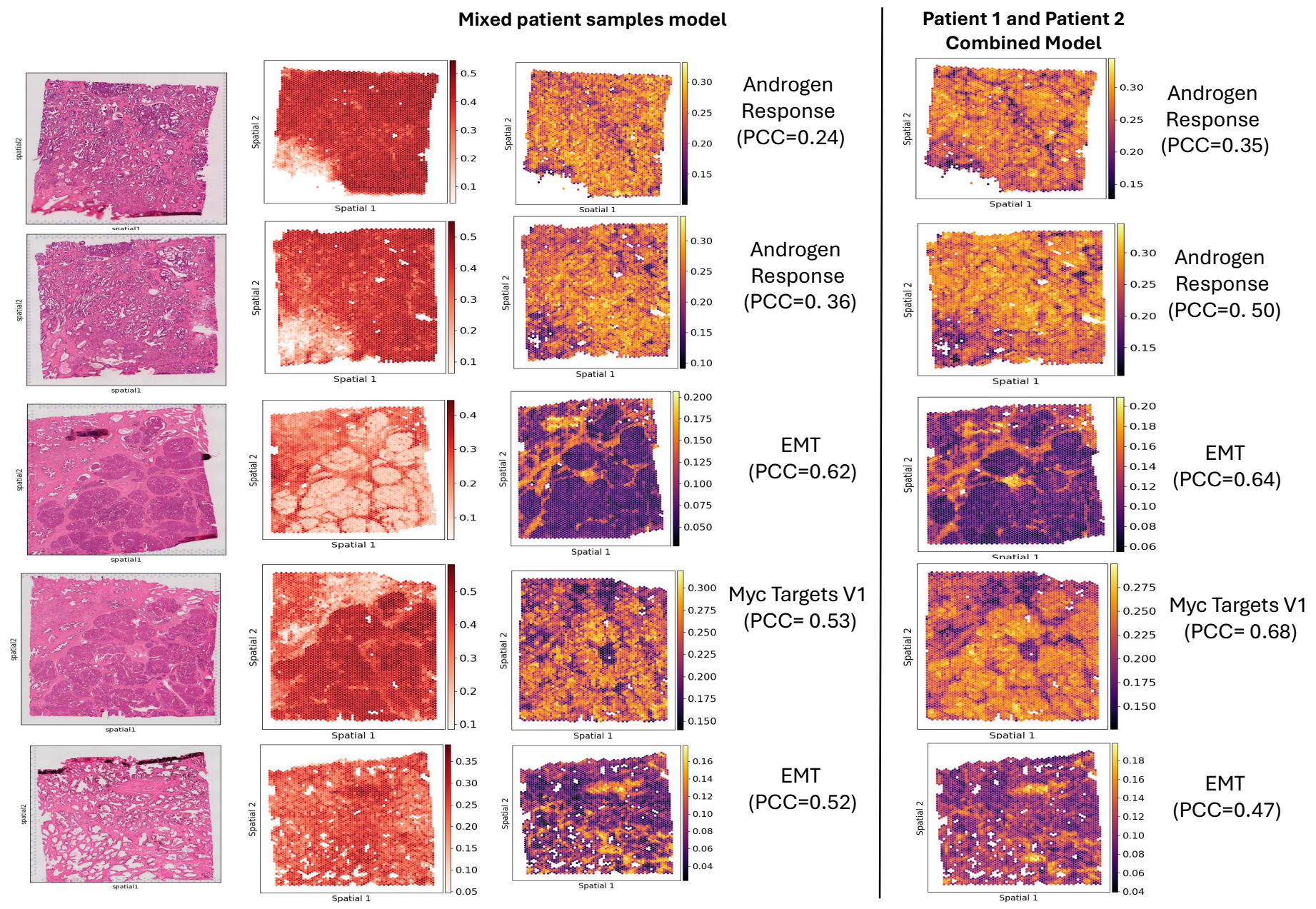

Supplementary Fig. 15: Evaluation of DeepPathway model using small prostate data as an independent data cohort where the model is trained (i) using mixed patient samples of P1 and P2 **(left)** and (ii) using model trained on all samples of P1 and P2 (**(right)**) for showing its robustness and generalizability.

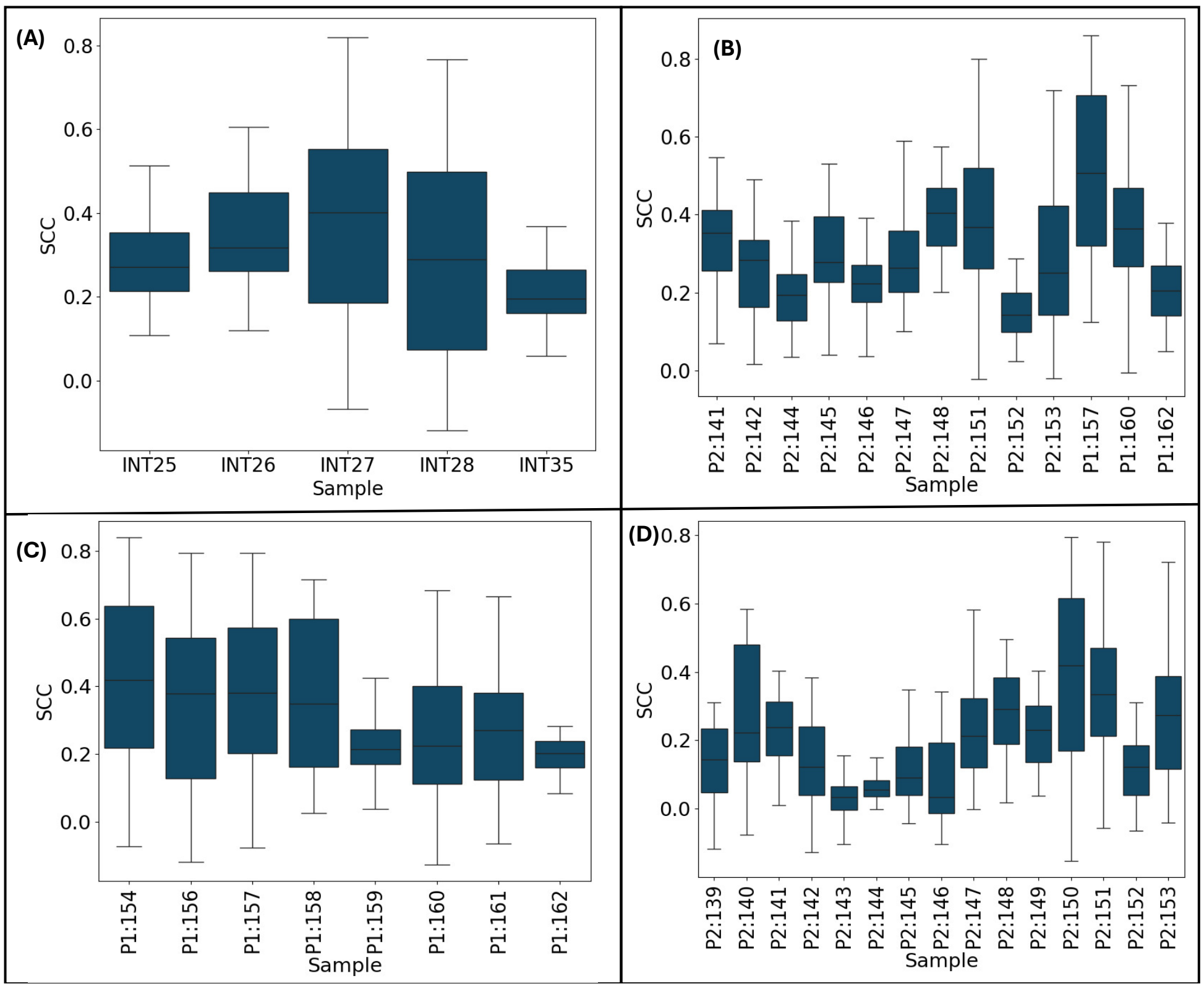

Supplementary Fig. 16: Spearman Correlation coefficient (SCC) distribution results of DeepPathway under different experimental settings. **(A)** Small prostate cancer dataset. **(B)** mixed patients' samples approach **(C)** Patient 1 is evaluated on trained model of Patient 2 samples. **(D)** Patient 2 is evaluated on trained model of Patient 1 samples.

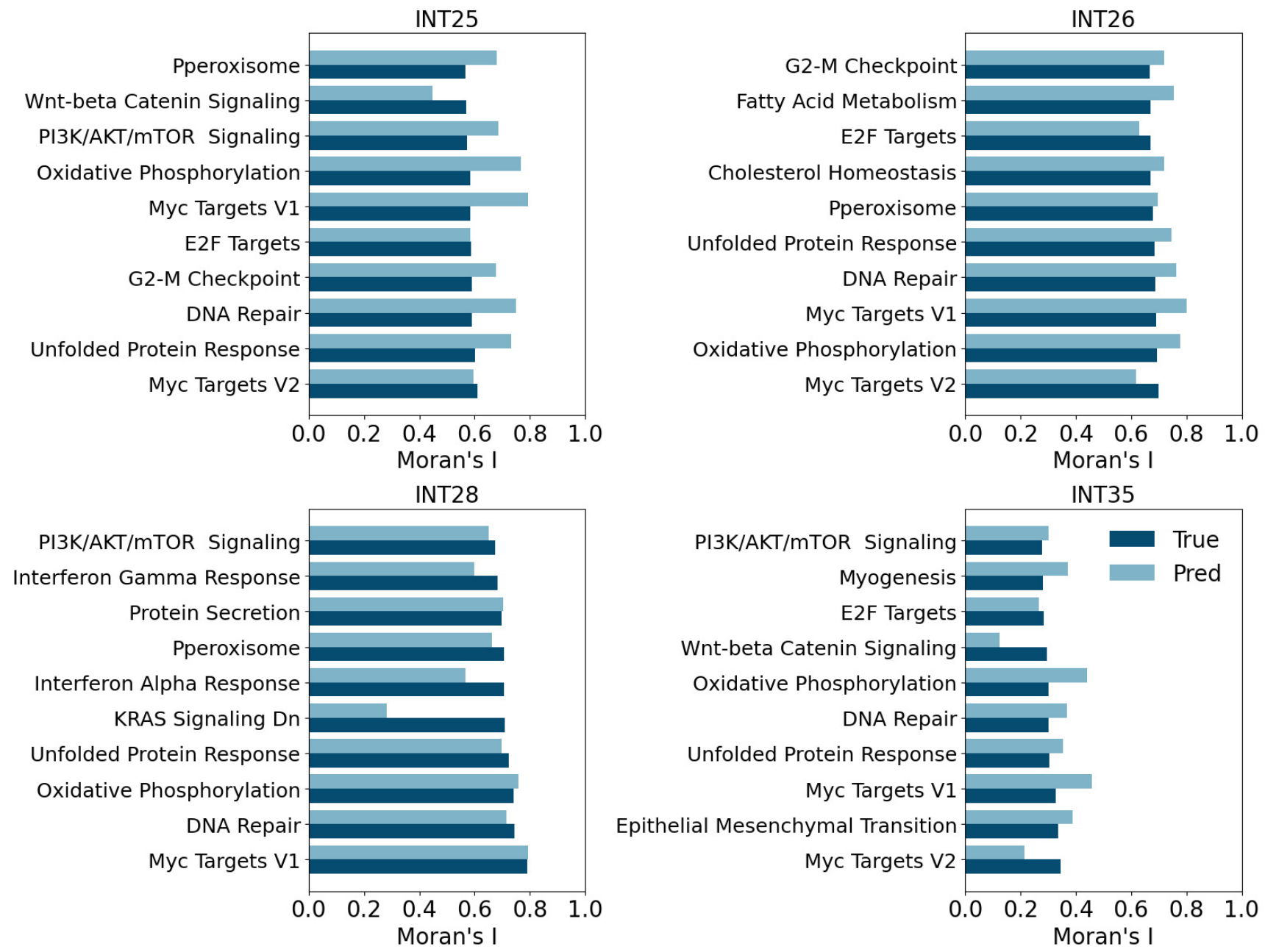

Supplementary Fig. 17: Moran's I showing the spatial autocorrelation of top 10 pathways (selected based on highest autocorrelation valued on ground truth) for small prostate cancer dataset.

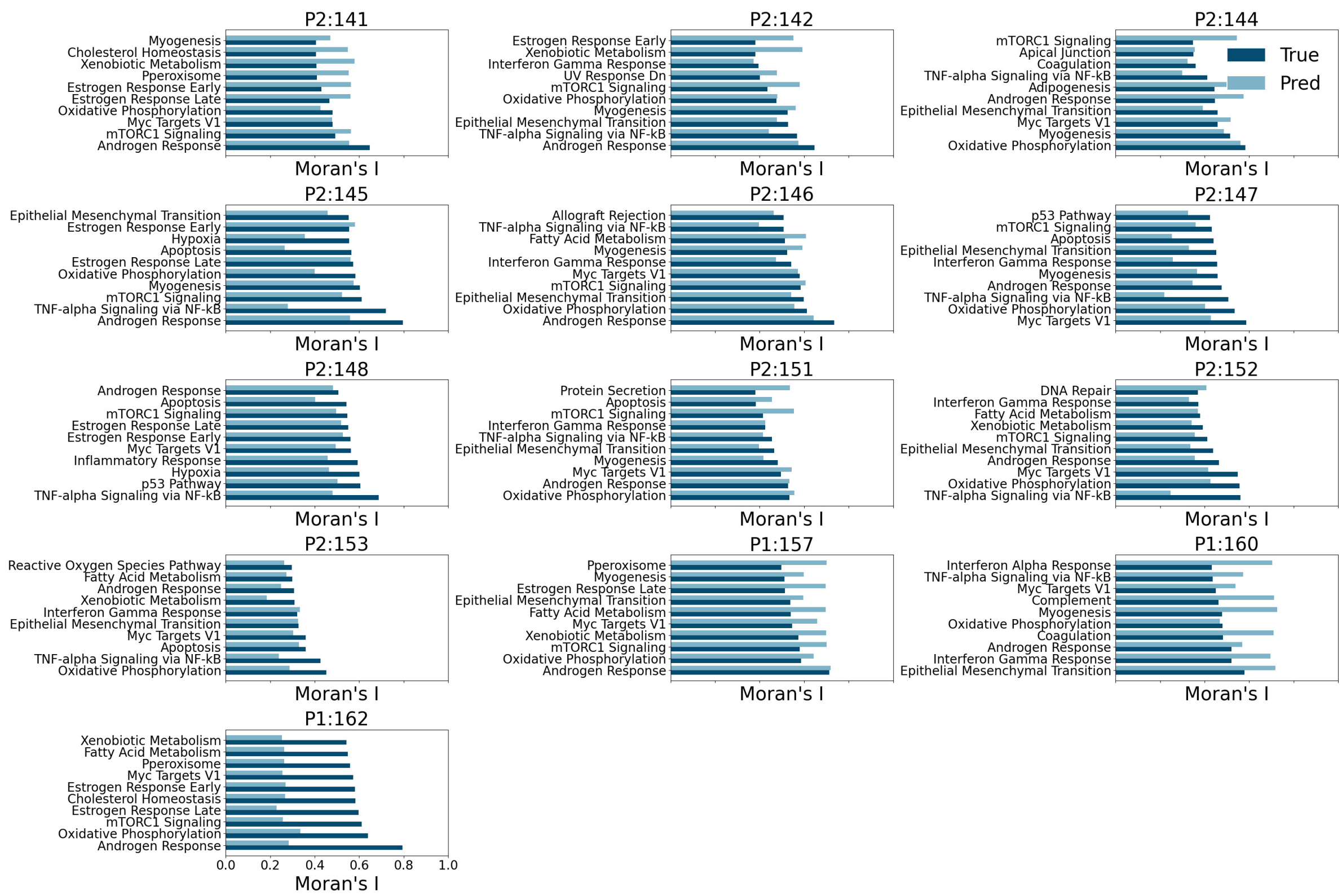

Supplementary Fig. 18: Moran's I showing the spatial autocorrelation of top 10 pathways (selected based on highest autocorrelation valued on ground truth) for all test samples of P1 when DeepPathway is trained on mixed patients' s samples of P1& P2.

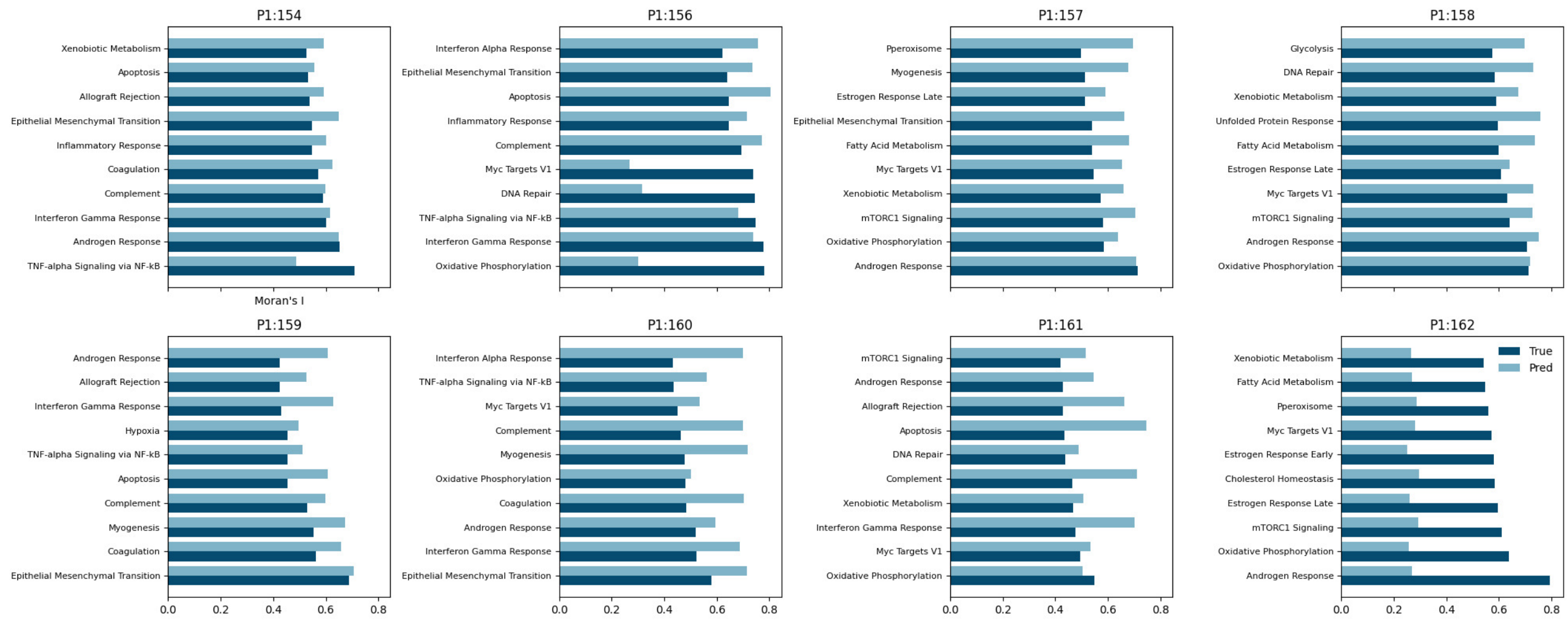

Supplementary Fig. 19: Moran's I showing the spatial autocorrelation of top 10 pathways (selected based on highest autocorrelation valued on ground truth) for all test samples of P1 when DeepPathway is trained on samples of P2 only.

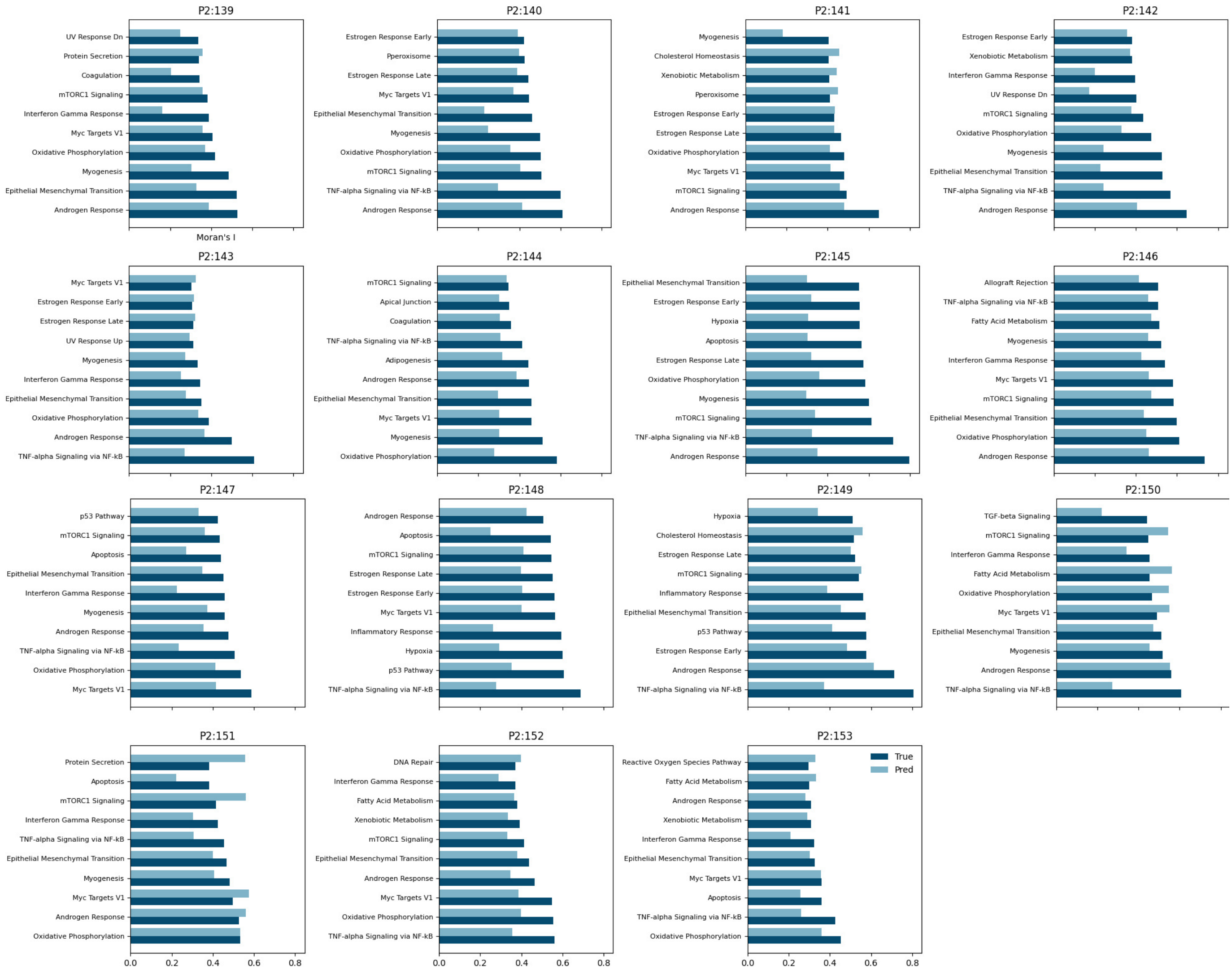

Supplementary Fig. 20: Moran's I showing the spatial autocorrelation of top 10 pathways (selected based on highest autocorrelation valued on ground truth) for all test samples of P2 when DeepPathway is trained on samples of P1 only.

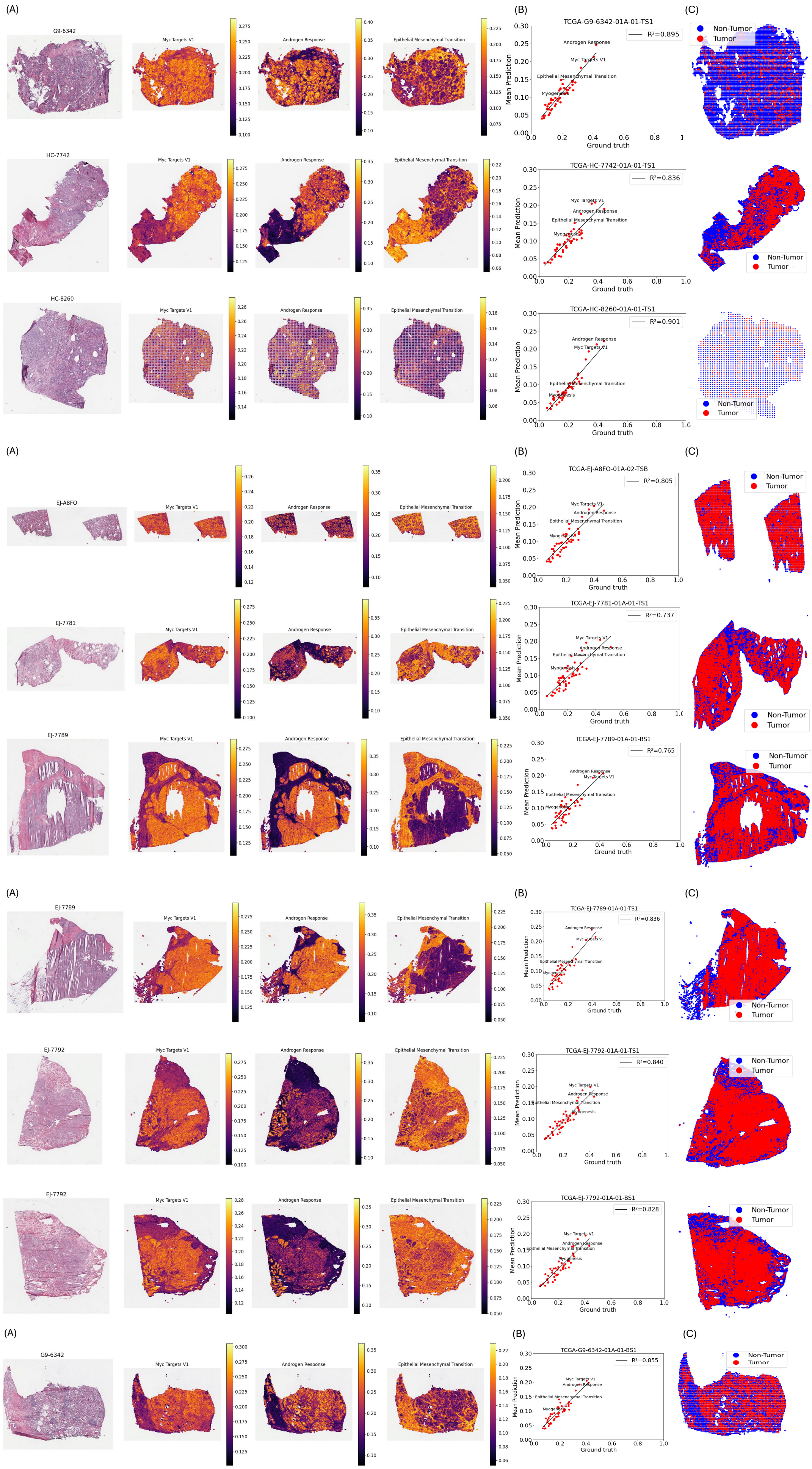

Supplementary Fig. 21: (A) H&E and spatial visualization obtained from the DeepPathway model for MyC Targets V1, Androgen Response, and Myogenesis on TCGA prostate tumor patients. (B) A scatter plot showing the quantitative results computed between bulk RNA-seq data and mean prediction. (C) Orthogonal validation with patch-wise tumor classification from the model trained on the TCGA prostate cancer dataset.

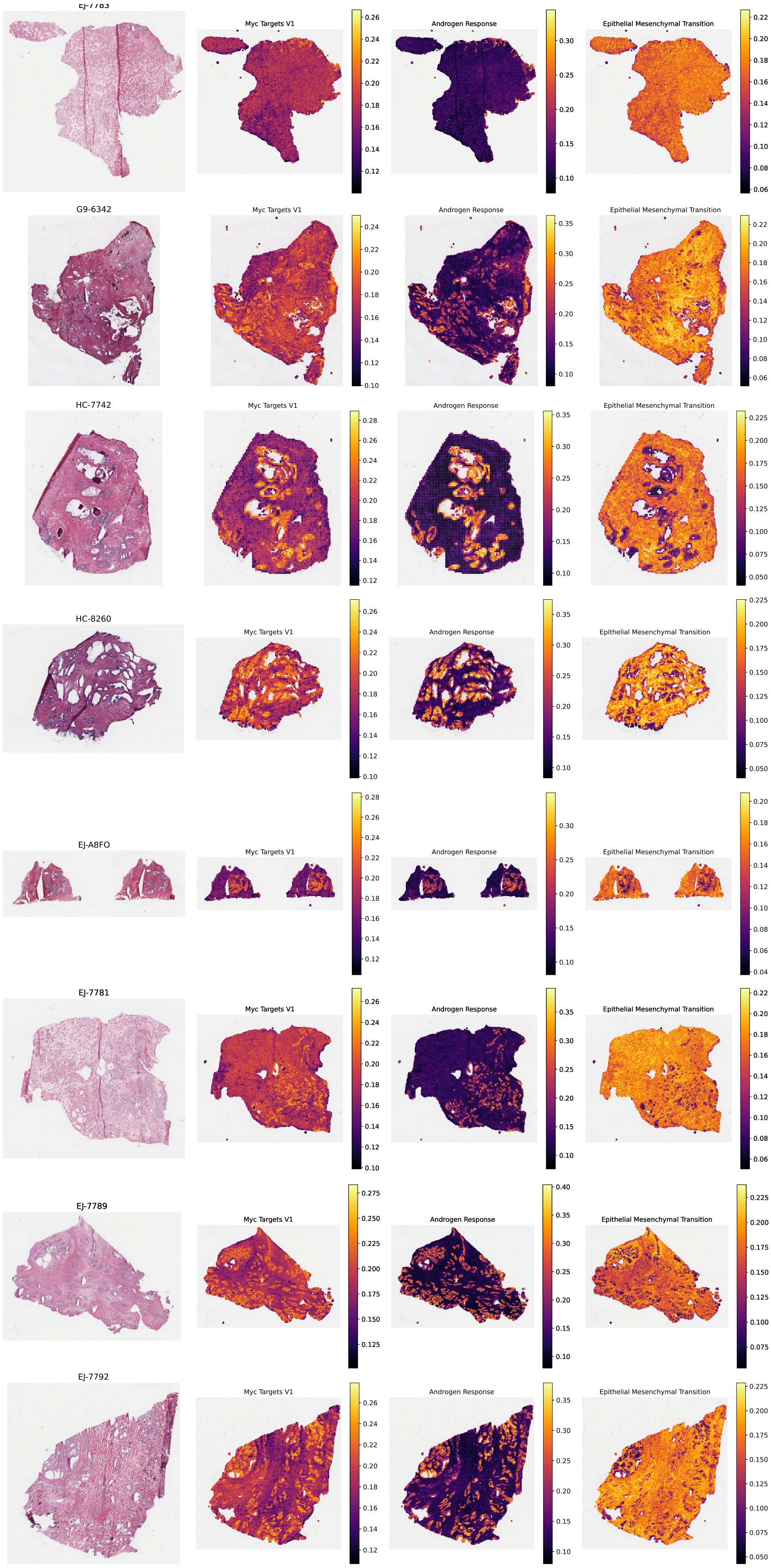

Supplementary Fig. 22: Visualization of Normal TCGA prostate H&E slides.

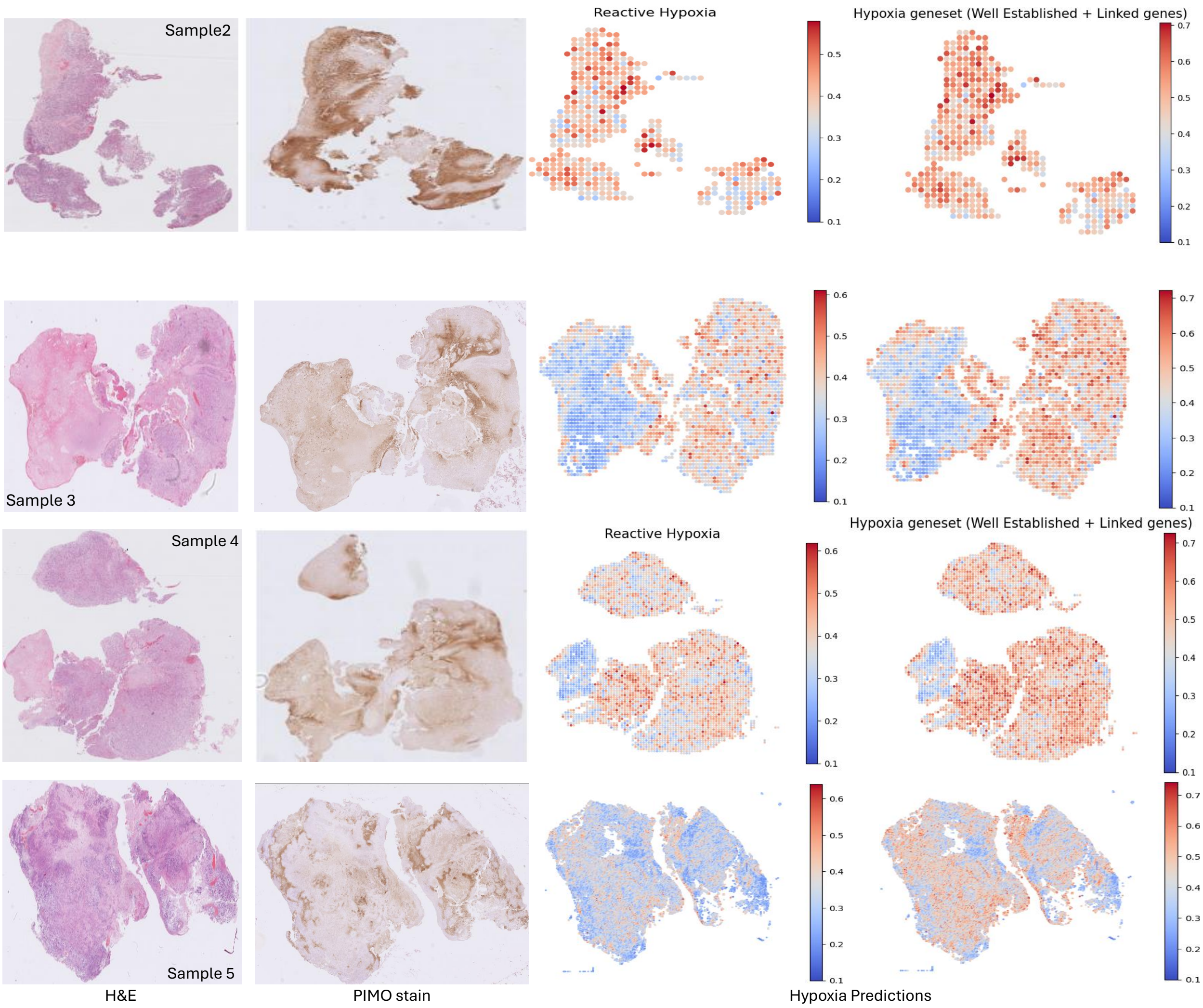

Supplementary Fig. 23: Visualization of H&E images, PIMO stainin g validation, and predicted Reactive Hypoxia pathway and a custom hypoxia geneset comprised of well-established hypoxic genes along with their linked genes from H&Es of brain tumour patients (row-wise) using DeepPathway.
